# Mechanism of the natural product moracin-O derived MO-460 and its targeting protein hnRNPA2B1 on HIF-1α inhibition

**DOI:** 10.1038/s12276-018-0200-4

**Published:** 2019-02-12

**Authors:** Nak-Kyun Soung, Hye-Min Kim, Yukihiro Asami, Dong Hyun Kim, Yangrae Cho, Ravi Naik, Yerin Jang, Kusic Jang, Ho Jin Han, Srinivas Rao Ganipisetti, Hyunjoo Cha-Molstad, Joonsung Hwang, Kyung Ho Lee, Sung-Kyun Ko, Jae-Hyuk Jang, In-Ja Ryoo, Yong Tae Kwon, Kyung Sang Lee, Hiroyuki Osada, Kyeong Lee, Bo Yeon Kim, Jong Seog Ahn

**Affiliations:** 1 Anticancer Agent Research Center, Korea Research Institute of Bioscience and Biotechnology, Cheongju, 28116 Korea; 2 Department of Biomolecular Science, University of Science and Technology, Daejeon, 34113 Korea; 3 Chemical Biology Research Group, RIKEN CSRS, Wako, Saitama, 351-0198 Japan; 4 College of Pharmacy, Dongguk University, Goyang, 10326 Korea; 5 Protein Metabolism Medical Research Center, Department of Biomedical Sciences, College of Medicine, Seoul National University, Seoul, 03080 Korea; 6 Lab of Metabolism, National Cancer Institute, National Institutes of Health, Bethesda, MD 20892 USA; 7 Present Address: Kitasato Institute for Life Sciences, Kitasato University, 5-9-1 Shirokane, Minato Ku, Tokyo 108-8641 Japan

**Keywords:** Targeted therapies, Targeted therapies, Targeted therapies, Targeted therapies

## Abstract

Hypoxia-inducible factor-1α (HIF-1α) mediates tumor cell adaptation to hypoxic conditions and is a potentially important anticancer therapeutic target. We previously developed a method for synthesizing a benzofuran-based natural product, (R)-(-)-moracin-O, and obtained a novel potent analog, MO-460 that suppresses the accumulation of HIF-1α in Hep3B cells. However, the molecular target and underlying mechanism of action of MO-460 remained unclear. In the current study, we identified heterogeneous nuclear ribonucleoprotein A2B1 (hnRNPA2B1) as a molecular target of MO-460. MO-460 inhibits the initiation of HIF-1α translation by binding to the C-terminal glycine-rich domain of hnRNPA2B1 and inhibiting its subsequent binding to the 3’-untranslated region of *HIF-1α* mRNA. Moreover, MO-460 suppresses HIF-1α protein synthesis under hypoxic conditions and induces the accumulation of stress granules. The data provided here suggest that hnRNPA2B1 serves as a crucial molecular target in hypoxia-induced tumor survival and thus offer an avenue for the development of novel anticancer therapies.

## Cancer: How a plant metabolite analog suppresses tumor growth

A synthetic analog of a chemical found in fruit suppresses tumor growth by targeting an RNA-binding protein (hnRNPA2B1) and preventing the production of a pro-cancer regulatory factor. Nak-Kyun Soung from the Korea Research Institute of Bioscience and Biotechnology, Cheongju, South Korea, and coworkers built on their previous discovery that a compound derived from a medicinal plant metabolite can suppress the activity of hypoxia-inducible factor-1α (HIF-1α). This protein, which is involved in many aspects of cancer biology, is activated in the low-oxygen microenvironments found inside tumors. The researchers show that the compound binds to a protein that helps with the conversion of HIF-1α–encoding RNA transcripts into HIF-1α proteins. Liver cancer cells treated with the compound grew slowly and produced less HIF-1α under both normal and low-oxygen culture conditions, highlighting the potential of this anti-cancer strategy.

## Introduction

Rapid proliferation of cells in solid tumors causes varying degrees of hypoxia in living tissue^1^. Cancer cells under hypoxic conditions accumulate a range of proteins through transcriptional induction, which promotes the growth of tumor tissue and provides resistance to cell death. These proteins play critical roles in angiogenesis, cell proliferation, glucose uptake, apoptosis, and metastasis^2–4^. The transcriptional induction of these genes is mediated by a master regulator, the transcription factor hypoxia-inducible factor (HIF)-1α^1^, and results in the selection of cancer cells that are adapted to hypoxic conditions. The presence of active HIF-1α and subsequent induction of its downstream genes also promote cancer cell resistance to chemotherapy through various cellular responses (e.g., suppressing apoptosis^5^, bypassing senescence^6,7^, altering cellular metabolism^8^, increasing drug efflux^9^). Thus, the inhibition of HIF-1α activity has attracted considerable interest by those looking to develop efficient therapeutic strategies for various solid cancers. The HIF-1 transcriptional complex is a heterodimeric protein composed of HIF-1α and HIF-1β subunits. The alpha subunit is continuously transcribed and translated and its protein stability is regulated by oxygen levels in the host tissue^2^. HIF-1 transcriptional complex activity is regulated by the stability of the alpha subunit, which accumulates under hypoxic conditions, but is rapidly degraded under normoxic conditions, whereas the levels of the beta subunit do not fluctuate. This accumulation of HIF-1α is a prerequisite for its activity.

A growing number of small molecules have been shown to inhibit HIF-1α activity through a wide variety of molecular mechanisms (e.g., a reduction in HIF-1α levels by inhibiting its synthesis or promoting its degradation, reductions in HIF-1 activity through impaired heterodimerization of the two subunits, reduced DNA binding, and decreased transactivation^10^). The small molecule geldanamycin promotes HIF-1α degradation independently of the canonical HIF-1α degradation pathway^11^. The small molecules PX-478 (HIF translation) and BAY-872243 inhibit HIF-1 activity and are under investigation in clinical trials for the treatment of cancer^12,13^. (R)-(-)-moracin-O, with its arylbenzofuran ring, is a natural product isolated from *Morus* species that exerts potent inhibitory effects on HIF-1α accumulation under hypoxic conditions^14^. The absolute configuration of naturally occurring (R)-(-)-moracin-O was previously determined and its first total synthesis was subsequently achieved^15^. A systematic analysis of the structure-activity relationship of (R)-(-)-moracin-O during that study led to the discovery of MO-460, i.e., (R)-4-[6-(1-hydroxy-1- parent compound^16^.

The objectives of the current study were to identify the molecular target(s) of MO-460 and to characterize the molecular mechanism of its inhibitory effect on HIF-1α; we used several approaches. These approaches included an affinity capture method followed by identification of putative target proteins using mass spectrometry, a chemical-protein binding assay, and conventional biological assays. We found that MO-460 did not directly interact with HIF-1α protein. Rather, it inhibited HIF-1α accumulation by interacting with the heterogeneous nuclear ribonucleoprotein A2/B1 (hnRNPA2B1), which was previously unknown in the regulatory pathways of HIF-1α synthesis. hnRNPA2B1 is a member of the hnRNP family of RNA binding proteins and plays key roles in multiple aspects of nucleic acid metabolism (e.g., alternative splicing^17,18^, mRNA trafficking^19^, telomere biogenesis^20,21^, and transcriptional and translational regulation^22,23^). HnRNPA2B1 is also involved in apoptosis and epithelial-to-mesenchymal transition (EMT)^24^. Moreover, it is overexpressed in several cancers, including glioblastoma, breast, and lung, and its expression level is positively correlated with poor prognosis^24,25^. Therefore, it is used as a new target for cancer therapy and a biomarker for cancer diagnosis^26,27^. Herein, the identification of this novel molecular target of MO-460 and its mode of action creates new potential avenues for cancer treatment. In addition, MO-460, a small molecule targeting HIF-1α under hypoxia, merits further development as an anticancer drug.

## Materials and methods

### Synthesis of MO-460 and its biotin conjugated form analogues (Biotin linked MO-460)

Please see online Supplementary Materials and Methods 1.

### Cell culture, antibodies, and siRNA transfection

Hep3B and HEK293Tcells were purchased from American Type Culture Collection (ATCC) (Manassas, VA) in April 2013. Cells were passaged for less than 2 months before resuscitation for this work. Cells were routinely tested for mycoplasma contamination using the e-Myco Mycoplasma PCR Detection Kit (iNtRon Biotech.). The last test was done in December 2016. All cell lines were revived every 2 to 3 months. Cells were cultured as recommended by the ATCC. Transfection was routinely carried out with HiPerFect (Qiagen). Hep3B cells (5 × 10^4^ cells/mL) were seeded in 12-well dishes and incubated for 12 h. The cells were then transfected with or without 200 pmol of siRNAs using HiPerFect and incubated for 48 h with or without 200 μM CoCl_2_. All antibodies used in this study are listed in Supplementary Materials and Methods 2.

### Plasmid construction

Detailed information on the construction of various plasmids and production of the lentivirus are described in the Supplementary Materials and Methods 3. All RNAi target products and sequences used in this study are listed in Supplementary Materials and Methods 4.

### Anti-hnRNPA2B1 antibody generation

Bacterial His-tagged hnRNPA2B1, purified as described above, was injected into BALB/c mice. Hybridomas were prepared by fusing spleen cells with cells of myeloma line SP2/0-Ag14 using previously described procedures^26^. Enzyme-linked immunosorbent assays (ELISA) were performed to insure that each monoclonal antibody selected reacted exclusively with hnRNPA2B1. The prepared antibodies were available for immunoblotting (IB), immunoprecipitation and immunocytochemistry (ICC).

### Detection of binding proteins for Biotin-MO-460

We synthesized biotinylated MO-460 using a recently reported method^15^. Fractionation and enrichment of cytosol and nuclei were performed as described previously^28^. Briefly, Hep3B (Human hepatocyte cancer cell line) was harvested and washed twice with PBS after treatment with 200 μM of CoCl_2_ for 24 h, and then resuspended in lysis buffer [10 mM HEPES pH 7.9, 10 mM KCl, 0.1 mM EGTA, 0.1 mM EDTA, 0.5 mM PMSF, 0.025% 2-Mercaptoethanol, 1.6% NP-40, and protease inhibitor cocktail]. After cell lysis and homogenization by vortexing for 10 s, the insoluble material was removed by centrifugation. The supernatant was collected as a cytosol-enriched lysate. The pellet was resuspended in nuclear extract buffer [20 mM HEPES pH 7.9, 400 mM NaCl, 1 mM EGTA, 1 mM EDTA, 1 mM DTT, 1 mM PMSF, protease inhibitor cocktail]. The pellet was then vortexed vigorously at 4 °C to separate the insoluble material. After the cytosol-enriched lysate (97.9 μg of protein) and the nuclei-enriched lysate (67.5 μg of protein) were precleared by incubation with NeutrAvidine^TM^ agarose-resins (Thermo Scientific) that were conjugated with biotin-linker (cytosol fraction: 600 μM, nuclear fraction: 1200 μM) for 12 h at 4 °C, the cleared cell lysate was incubated with NeutrAvidine^TM^ agarose-resins bound -Biotin-MO-460 (cytosol fraction: 200 μM, nuclear fraction: 400 μM) for 16 h at 4 °C, respectively. The reacted samples were washed three times with binding buffer [10 mM HEPES pH 7.9, 10 mM KCl, 0.1 mM EGTA, 0.1 mM EDTA, 0.5 mM PMSF, 0.025% 2-Mercaptoethanol, and protease inhibitor cocktail] and the bound proteins eluted with SDS-PAGE sample buffer. The proteins were then separated on a 4–20% SDS-polyacrylamide gradient gel and visualized using a silver staining kit (Thermo Scientific).

### Identification of binding proteins for Biotin-MO-460

To identify the binding proteins, we recovered prominent protein-bands from biotin-MO-460 regions and biotin-linker regions (same-sized areas) in the SDS-polyacrylamide gel. The bands were reduced in 1,4-dithiothreitol (10 mM), alkylated with iodoacetamide (50 mM), and then washed, dried, and rehydrated in trypsin solution (12 ng/μL) on ice for 1 h. After adding 20 μL of ammonium bicarbonate (50 mM), samples were digested overnight at 37 °C and the peptides extracted and dried. The dried peptides were rehydrated with a solution of 20 L of 0.1% formic acid and 2% acetonitrile (ACN). Eight microliters of the resulting peptide mixture were injected onto a nano acquity LC system (Waters Corp. Manchester, United Kingdom). The peptides were separated on a 1.7 μm, 75 μm × 150 mm BEH C-18 column (Waters Corp. Manchester, United Kingdom). The gradient (Solution A: 0.1% formic acid, solution B: 0.1% formic acid, 100% ACN) started at 5% and ended at 40% B after 45 min. MS and MS/MS data were acquired using a Q-TOF Premier mass spectrometer (Waters Corp., Micromass, Manchester, UK). Double- and triple-charged peptide ions were automatically selected by MassLynx software and fragmented. MS data were processed and peak lists for protein identifications by database searches were generated by PLGS software. Database searches were carried out with MASCOT server version 2.2.0 using the NCBInr protein database. The peptide tolerance was set at 30 ppm and the MS/MS tolerance to 0.1 Da.

### In vitro pull-down assay

HEK293T cells were cultured in DMEM medium supplemented with 10% fetal bovine serum in a humidified atmosphere containing 5% CO_2_. The cells were transfected with C-terminal Myc/DDK-tagged hnRNPA2B1 constructs. Recombinant protein was captured with an anti-DDK affinity column followed by conventional chromatography. A streptavidin-conjugated biotin linker was used as a control or streptavidin-conjugated biotinylated MO-460 were treated with 5% bovine serum albumin in PBS for 1 h at room temperature. Myc/DDK-tagged hnRNPA2B1 protein (1 ug) was incubated with streptavidin-conjugated biotin linker (1,200 μM) or streptavidin-conjugated biotinylated MO-460 (400 μM) in the presence or absence of MO-460 (400 μg) in binding buffer for 6 h at 4 °C. The reacted beads were washed with binding buffer [10 mM HEPES pH 7.9, 10 mM KCl, 0.1 mM EGTA, 0.1 mM EDTA, 0.5 mM PMSF, 0.025% 2-mercaptoethanol, and protease inhibitor cocktail] and the bound proteins eluted with SDS/PAGE sample buffer. Proteins were resolved by SDS/PAGE and detected by silver staining.

### Analysis of the interaction between MO-460 and the hnRNPA2B1 binding region using the optical sensor

The binding capacity of hnRNPA2B1 to Biotin-MO-460 was also analyzed using a BLItz™ (Fortebio, Inc., CA, US) instrument with a streptavidin-coated biosensor tip (Pall, USA). The streptavidin-coated tips were pre-wetted for 10 min in an incubation buffer (10 mM HEPES pH 7.4, 1% BSA and 0.05% Tween 20). The streptavidin-coated tips were immobilized with 1 μM Biotin-MO-460 in incubation buffer (10 mM HEPES pH 7.4, 1% BSA and 0.05% Tween 20) for 5 min. The biosensor was washed with HEPES (pH 7.4) and incubated with 250-, 500- and 1,000-nM concentrations of the full-length and truncated hnRNPA2B1 proteins for 3 min. The concentrations were then incubated with HEPES for another 3 min to desorb the protein. Analysis of the accurate and precise kinetic constants was performed by the BLItz data analysis software (BLItz Pro 1.1).

### Immunofluorescence staining for HIF-1α

For immunofluorescence observation, Hep3B cells were seeded on microplates at 2 or 5 × 10^4^ cells/mL and cultured for 18 h. The cells were further cultured either with or without 200 μM CoCl_2_ and simultaneously exposed to various concentrations of MO-460 or siRNAs for 48 h. The medium in the microplate wells was then removed and cells were fixed with 3.7% formaldehyde in PBS for 15 min then permeabilized for 5 min with 100% cold MeOH. Cells were washed with PBS containing 1% bovine serum albumin (PBS-1% BSA), and then treated with 5% bovine serum albumin for 20 min. The cells in each well were treated with HIF-1α antibody in PBS-0.1% BSA then placed in a humidified atmosphere of 5% CO_2_ at 37 °C and incubated for 60 min. After being washed with PBS-0.1% BSA, treated wells of HIF-1α were incubated with Alexa Fluor 488-conjugated mouse anti-IgG antibody in PBS-0.1% BSA then incubated at room temperature for 60 min. After being washed with PBS-0.1% BSA, the dish wells were overlaid with Hoechst 33342 for 5 min at room temperature and then washed with PBS-0.1% BSA. Fluorescence was photographed with an inverted ECLIPSE Ti-U microscope (Nikon Corporation, Japan).

### RNA extraction and semi-quantitative RT-PCR

Cells were lysed with TRIzol (Invitrogen) and proteins were extracted with chloroform. RNAs were precipitated with isopropanol and washed with 75% ethanol diluted with DEPC-treated water. Purity and concentration of RNA was analyzed using nano drop. The cDNA was synthesized from total RNA in a reaction mixture containing 5 × reaction buffer (250 mM Tris-HCl pH 8.3, 375 mM KCl, 15 mM MgCl_2_, 50 mM DTT), 50 μg/mL Oligo-dT, 10 mM dNTP, and 50,000 U M-MLV reverse transcriptase (Promega, Madison, WI). Amplified PCR products obtained with β actin-specific primers served as internal controls. The sequences of the primers used for this study are listed in Supplementary Materials and Methods 5 and 6.

### Purification of hnRNPA2B1 interacting with RNA

Hep3B cells were cultured at 37 °C in a 5% atmospheric concentration of CO_2_ and 200 μM of CoCl_2_ in the presence or absence of MO-460 (50 μM) and the cells harvested after 24 h. Total lysates of Hep3B were prepared in nuclear isolation buffer [128 mM sucrose, 40 mM Tris-HCl pH 7.5, 20 mM MgCl_2_, 4% Triton-X100]. After clarifying by centrifugation at 2500×* g* for 15 min at 4 °C the nuclear pellet was resuspended in RIP buffer [150 mM KCl, 25 mM Tris-HCl pH 7.5, 5 mM EDTA, 0.5 mM DTT, 0.5% Triton-X100, and protease inhibitor cocktail], and then incubated with 4 μg of either control IgG or the anti-hnRNPA2B1 antibody. After 1.5 h of incubation, protein-G agarose beads (Santa Cruz Biotechnologies, Santa Cruz, CA) were added and then precipitated. RNAs were purified from 90% of the samples. Eluted RNAs were complemented by reverse transcriptase with random primers. cDNAs were used in the subsequent PCR reactions with the indicated primer sets. The resulting PCR products were separated on a 1.5% agarose gel. The other 10% of the samples from which RNA was not collected were separated by SDS-PAGE and then immunoblotted with anti-hnRNPA2B1 antibody.

### In vitro translation assay

HIF-1α proteins were synthesized using in vitro transcription/translation systems (Promega, Madison, WI), the protocol was followed the manufacturer’s instructions. In brief, mixtures containing reaction components were incubated at 30 °C for 60 min, and then analyzed by Western blotting using HIF-1α. The template was used HIF-1α plasmid DNA containing untranslated region (Korea human gene bank, Daejeon, Rep. of Korea), and full-length recombinant hnRNPA2B1 and MO-460 were added to the mixture.

## Results

### Affinity capture reveals hnRNPA2B1 as target of MO-460

**Fig. 1:**
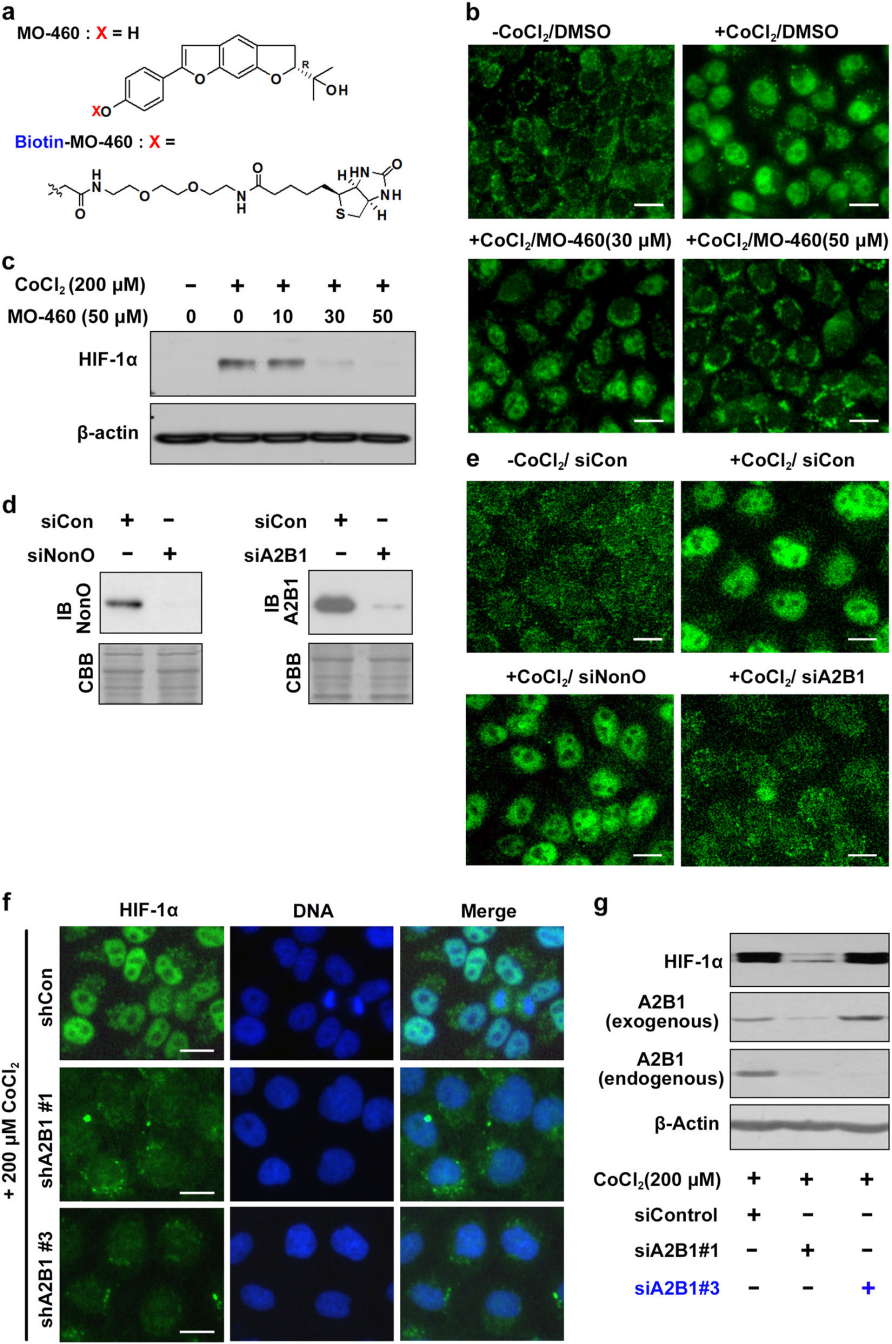
Identification of MO-460-binding proteins. **a** Structure of MO-460 and its chemical probe (biotin-MO-460). **b**, **c** Immunostaining and western blotting of HIF-1α with MO-460 under mimetic hypoxia (Cobalt chloride for 24 h). **d**, **e** Immunostaining and western blotting of MO-460 binding candidates in nuclear fractions under mimetic hypoxia. Knockdown in each batch of cells was confirmed by western blotting with the indicated antibodies. Equal protein loading was confirmed with Coomassie brilliant blue (CBB) staining; Immunostaining with anti-HIF-1α antibody. **f** Anti-HIF-1α immunostaining of Hep3B cells transduced with shRNAs targeting hnRNPA2B1 (shA2B1#1 and shA2B1#3) under mimetic hypoxia. **g** Rescue experiments for hnRNPA2B1 on HIF-1α accumulation. Cell lines constitutively expressing a GFP-hnRNPA2B1 fusion protein were treated with either siA2B1#1 targeting the coding region or siA2B1#3 targeting the 3’-untranslated region (UTR) of the hnRNPA2B1 gene. Scale bar, 20 μm

To determine the inhibitory mechanisms of MO-460, we synthesized MO-460 (or biotin-MO-460) (Fig. 1a) as the derivatives of Moracin-O which was reported previously^15^, and note that MO-460 inhibits the accumulation of HIF-1α protein under cobalt chloride (CoCl_2_)-derived mimetic hypoxic conditions in a concentration-dependent manner (Fig. 1b and c). For convenience, we used CoCl_2_ treatment to create hypoxic conditions in subsequent experiments and will refer to this as “mimetic hypoxia,” unless stated otherwise.

Next, an affinity capture approach was used to identify MO-460-interacting proteins in the nuclear and cytosolic fractions following the sequential pull-down assay and LC/MS analysis (Supplementary Figure S1a). Several hundred short peptide sequences had matched with various probabilities in protein databases (Supplementary Table S1). Of these, we selected 12 protein candidates with the highest affinities for MO-460 (Table 1 and Supplementary Figure S1b), and inhibited their expression using siRNA to determine their effects on HIF-1α accumulation in the nuclei under mimetic hypoxia (Fig. 1d and Supplementary Figure S2a). As a result, excluding hnRNPA2B1, a reduction in the expression of the 11 other genes (cytosolic fraction and NonO in nucleus fraction) did not affect nuclear localization of HIF-1α when examined by immunofluorescence. On the other hands, knockdown of hnRNPA2B1 led to a reduction of HIF-1α protein and nuclear localization (Fig. 1e and Supplementary Figure S2b). Interestingly, the depletion of HSP90β by siRNA caused a reduction in the levels of HIF-1α and prominent cell death, however, HIF-1α protein, like other binding candidates, localized to the nucleus (Supplementary Figure S2c).

**Table 1:** MO-460 binding candidates from mass spectrometry analysis

No.	UniPortKB	Protein name (Symbol)	Seq. coverage by Mascot report (%)
1	P04075	Aldolase A	39
2	P04406	GAPDH	39
3	Q15233	Non-POU Domain Containing, Octamer-Binding (NonO)	38
4	P08238	HSP90 β	38
5	O75607	Nucleophosmin 3 (NPM3)	35
6	P09651	hnRNPA1	32
7	P19338	nucleolin	34
8	P22626	hnRNPA2B1	33
9	Q6PJY1	DNA helicase V (FUBP1)	30
10	P55072	Valosin-Containing Protein (VCP)	23
11	Q92841	DDX17	<20
12	P31943	hnRNPH1	<20

To further confirm the observed results, we also knocked down hnRNPA2B1 using lentiviral shRNA. We generated three shRNAs targeting either the coding region (shA2B1 #1 and #2) or the 3’-untranslated region (UTR) (shA2B1 #3) of *hnRNPA2B1* mRNA (Supplementary Figure S2d), and observed inhibition of HIF-1α when hnRNPA2B1 levels were sufficiently reduced by shA2B1 #1 or #3 (Fig. 1f). We further tested if exogenous green fluorescent protein (GFP)-hnRNPA2B1 could compensate for the loss of endogenous hnRNPA2B1 by comparing the effects of siA2B1 #1 and siA2B1 #3 on HIF-1α accumulation. shA2B1 #1 (targeting the coding region) resulted in a severe reduction in both endogenous hnRNPA2B1 and GFP-hnRNPA2B1, leading to a dramatic reduction in HIF-1α protein level. In contrast, shA2B1 #3 (targeting the 3’-UTR) led to a reduction in hnRNPA2B1, but not GFP-hnRNPA2B1, which lacks the 3’-UTR and results in the accumulation of HIF-1α (Fig. 1g). These data indicate that knockdown of hnRNPA2B1 can be complemented by exogenous hnRNPA2B1, and suggest that hnRNPA2B1 is necessary for the accumulation of HIF-1α.

We then confirmed that real hypoxia (ie, a 1% oxygen concentration) caused an accumulation of HIF-1α, similar to that observed with CoCl_2_ treatment. Under real hypoxia, (R)-(-)-moracin O treatment, MO-460 treatment, and hnRNPA2B1 knockdown led to a reduction in HIF-1α accumulation (Supplementary Figure S3). These findings demonstrate for the first time that hnRNPA2B1 plays an important role in HIF-1α accumulation under mimetic hypoxia and that MO-460 can efficiently inhibit HIF-1α expression through hnRNPA2B1.

### MO-460 directly binds to the C-terminus of hnRNPA2B1

**Fig. 2:**
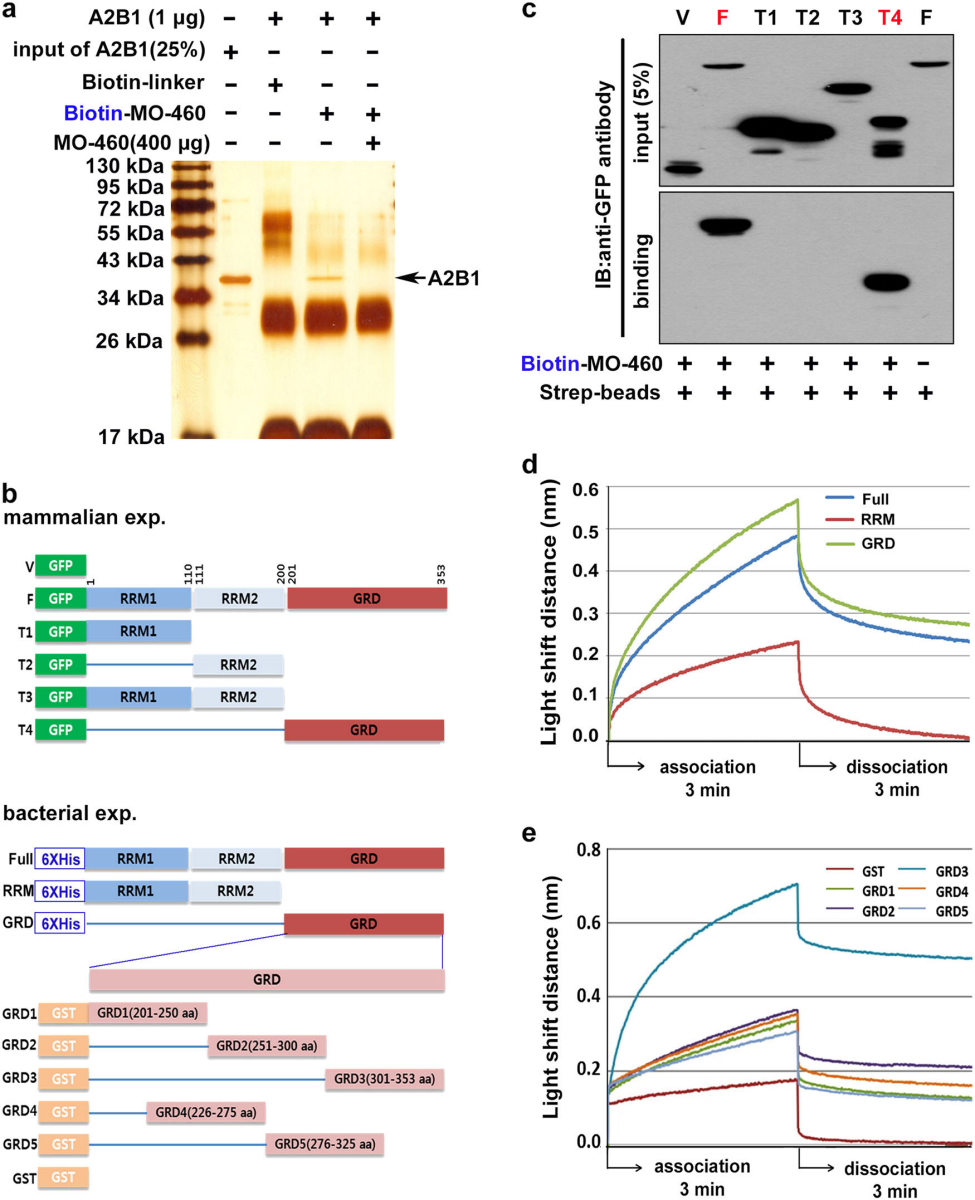
Direct interactions between MO-460 and hnRNPA2B1. **a** Pull-down assay of hnRNPA2B1 protein with biotin-MO-460 or in the presence of excess amounts of MO-460. **b** Schematic of hnRNPA2B1 protein construction variants. **c** Western blotting of GFP pull-down assay from cells transfected with varying constructs following incubation with streptavidin-conjugated biotinylated MO-460 (Biotin-MO-460); immunoblotting with anti-GFP antibody. **d**, **e** Kd values of MO-460 with three domain-dependent hnRNPA2B1 constructs and GRD domain constructs. Measurements were made with the BLITz^®^ system under sequential association and dissociation conditions. Binding affinity was measured every second for 3 min

Because hnRNPA2B1 in cell lysates was captured by affinity to MO-460 (Supplementary Figure S1), it was necessary to determine whether MO-460 binds directly to hnRNPA2B1. To perform pull-down assays, purified hnRNPA2B1 recombinant proteins were co-precipitated with biotin-MO-460. The interaction between hnRNPA2B1 and the biotin-MO-460 was eliminated by adding an excess amount of MO-460 to the assay mixture (Fig. 2a). These results suggest direct interactions between MO-460 and hnRNPA2B1.

We further established the MO-460 binding region on hnRNPA2B1 using stable cell lines that express domain-based constructs of hnRNPA2B1 fused to GFP (Fig. 2b, upper panel). In pull-down assays, both the full-length hnRNPA2B1 and the C-terminal glycine-rich domain (GRD) strongly bound to biotin-MO-460. However, the RNA recognition motifs RRM1, RRM2, and RRM1-RRM2 in the N-terminal half of the protein did not bind to biotin-MO-460 (Fig. 2c). Next, we measured MO-460 binding affinity to hnRNPA2B1 using the BLITz^®^ association-dissociation assay system. Purified bacterial recombinant proteins (Fig. 2b, bottom panel, and Supplementary Figure S4a) were attached to biotin-MO-460, and evaluated dissociation constants (Kd). The full-length hnRNPA2B1 and GRD clearly bound to biotin-MO-460 with apparent Kd of 1.14 × 10^−5^ M and 6.6 × 10^−6^ M, respectively, and the RRM of hnRNPA2B1 showed no specific interactions (Fig. 2d and Table 2 upper panel). Together, these experiments reveal that the C-terminal GRD of hnRNPA2B1 is critical for MO-460 activity.

**Table 2:** Binding affinities of MO-460 to the hnRNPA2B1 GRD region

hnRNPA2B1 GRD domain	Region (amino acid)	Con. (*u*M)	Kd (M)	Standard deviation (M)	Standard error (M)
Full*	1–353 aa	5	1.14 × 10^−5^	±1.57 × 10^−6^	±1.11 × 10^−6^
RRM*	1–193 aa	5	1.36 × 10^−2^	±4.21 × 10^−3^	±2.98 × 10^−3^
GRD*	202–353 aa	5	6.60 × 10^−6^	±2.15 × 10^−6^	±1.52 × 10^−6^
GST	-	5	5.00 × 10^−3^	±4.21 × 10^−4^	±2.10 × 10^−4^
GRD1	201–250 aa	5	3.05 × 10^−3^	±2.15 × 10^−3^	±1.07 × 10^−3^
GRD2	251–300 aa	5	3.67 × 10^−3^	±2.78 × 10^−3^	±1.39 × 10^−3^
GRD3	301–353 aa	5	2.99 × 10^−7^	±2.67 × 10^−7^	±1.34 × 10^−7^
GRD4	226–275 aa	5	3.48 × 10^−3^	±4.11 × 10^−3^	±2.05 × 10^−3^
GRD5	276–325 aa	5	3.35 × 10^−3^	±5.02 × 10^−3^	±2.51 × 10^−3^

* 6XHis tagged protein

To determine the binding site within the GRD, we designed five constructs that expressed ~50-amino-acid-long oligopeptides representing different regions within the GRD domain fused to glutathione-S-transferase (GST) (Fig. 2b, bottom panel, and Supplementary Figure S4b). As shown in the lower panel of Table 2, the Kd value between MO-460 and GRD3 was ~2.99 × 10^−7^ M (greater than 10^4^ times lower than that of the other GRDs under the same conditions). Specifically, only GRD3 containing 28 amino acids (amino acids 326–353) at the C-terminus strongly interacted with MO-460 (Fig. 2e and Table 2 bottom panel). The results of all assays consistently suggested that the two molecules interacted, indicating that MO-460 directly binds to hnRNPA2B1 through interaction with amino acids 326–353 in the C-terminal region.

### MO-460 does not affect *HIF-1α* transcriptional regulation

**Fig. 3:**
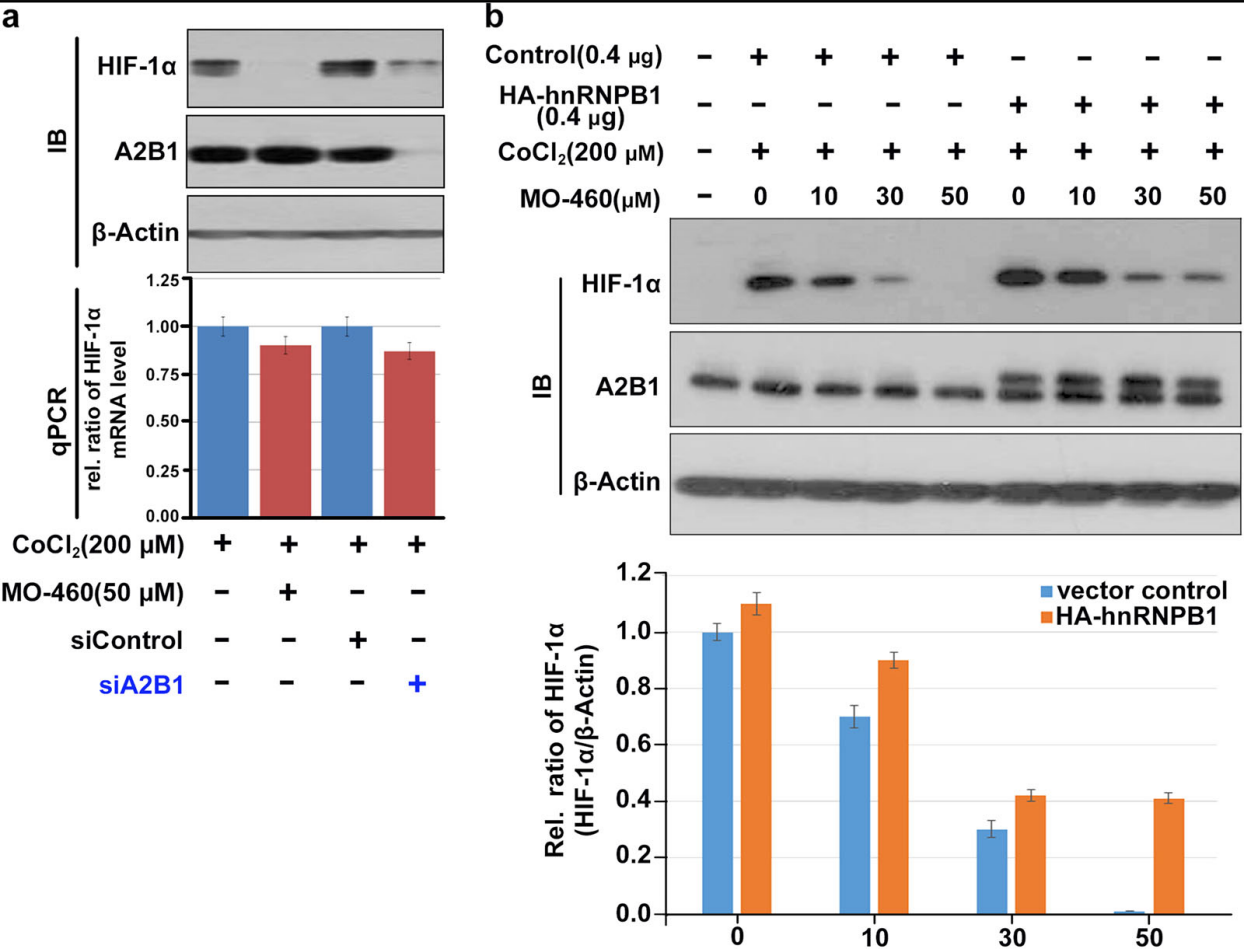
Interactions between MO-460, hnRNPA2B1, and HIF-1α. **a** Western blotting and qRT-PCR of HIF-1α were performed in Hep3B cells treated with MO-460 or hnRNPA2B1 siRNA under mimetic hypoxia. **b** Western blotting of HIF-1α was examined on MO-460 treatment in presence or absence of exogenous hnRNPA2B1. Data presented as the mean ± SD of three experiments

We evaluated the possibility that binding of MO-460 to hnRNPA2B1 regulates hypoxia-induced HIF-1α expression at the transcriptional level. As shown in Fig. 3a, treatment of Hep3B cells with 50 µM of MO-460 caused a marginal reduction (~10%) in *HIF-1α* mRNA level as detected with qRT-PCR. Treatment of MO-460 under normoxic condition had no observed effect (Supplementary Figure S5). In support of this result, knockdown of hnRNPA2B1 by siA2B1 also resulted in only a slight reduction in *HIF-1α* mRNA level. This slight reduction cannot account for the nearly complete elimination of HIF-1α proteins (Fig. 3a), suggesting posttranscriptional or translational control of HIF-1α expression by hnRNPA2B1 in response to MO-460.

MO-460 reduced the level of HIF-1α protein in a concentration-dependent manner with no effect on the amount of hnRNPA2B1. Exogenous overexpression of HA-hnRNPB1, however, induced a notable reversal of the inhibitory effect of MO-460 on HIF-1α accumulation, as revealed by western blot analysis (Fig. 3b). This indicates that the reduction in HIF-1α expression by MO-460 can be partially reversed by transient overexpression of hnRNPA2B1.

### MO-460 inhibits interaction between hnRNPA2B1 and the 3’UTR of *HIF-1*α mRNA

hnRNPA2B1 is an RNA-binding protein that binds to and regulates the stability or translation of target mRNAs^17,29^, and some RNA binding proteins are related to the regulation of *HIF-1α* mRNA turnover and translation^30,31^. To determine if hnRNPA2B1 binds to *HIF-1α* mRNA, we performed an RNA-immunoprecipitation assay. These result show that hnRNPA2B1 interacts with *HIF-1α* mRNA in Hep3B cell lysates or GFP-hnRNPA2B1 overexpression cell lysates (Supplementary Figure S7). However, there appears to be no direct interaction between HIF-1α protein and hnRNPA2B1, and MO-460 did not bind to HIF-1α protein in two rounds of the affinity capture experiment (Supplementary Figure S7 and Supplementary Table S1). Thus, these data suggest that hnRNPA2B1 binds to *HIF-1α* mRNA, not HIF-1α protein.

**Fig. 4:**
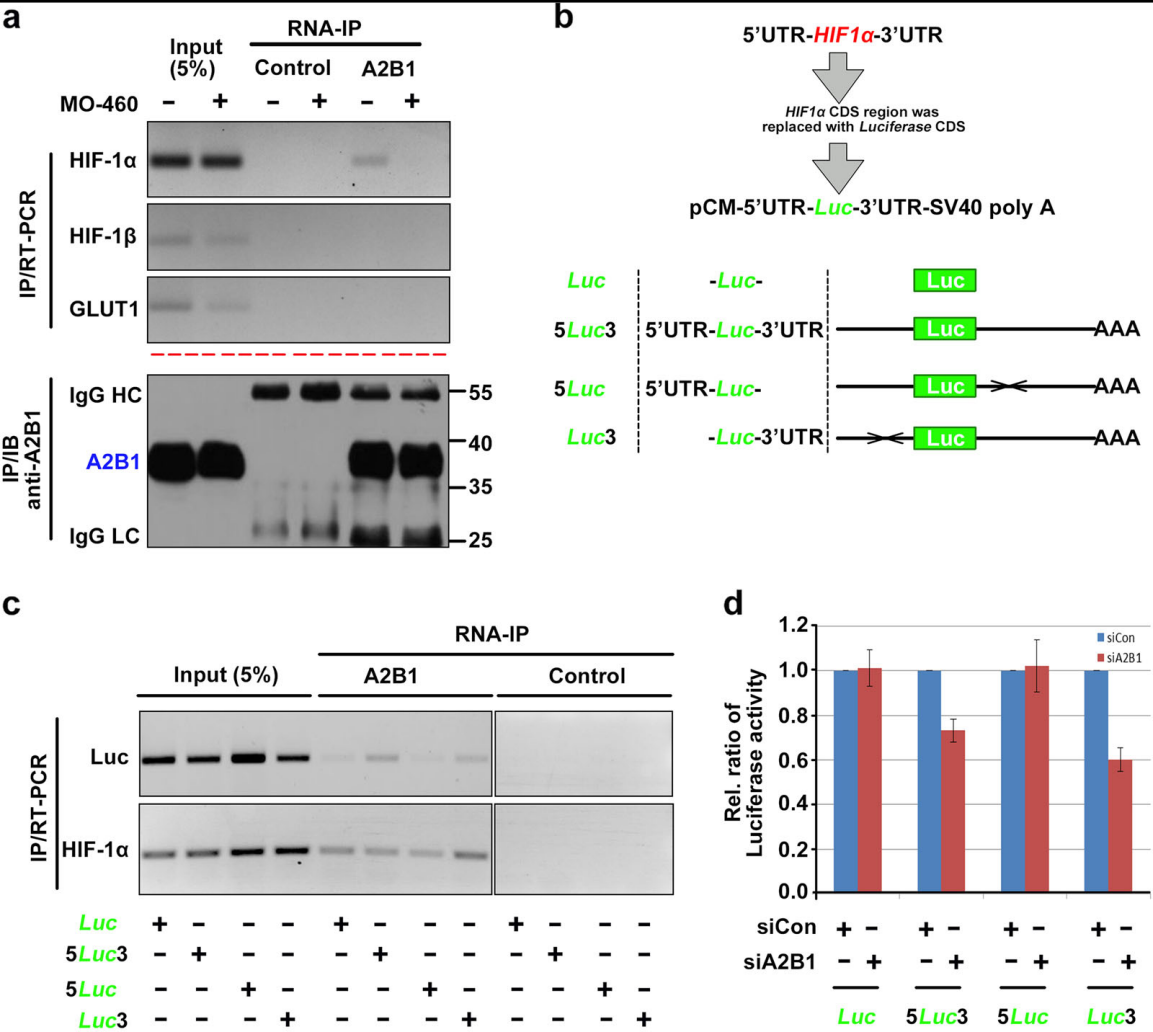
Inhibitory effect of MO-460 mediated by direct interaction of hnRNPA2B1 with the 3’-UTR of *HIF-1α* mRNA. **a** Effect of MO-460 on RNA-immunoprecipitation with hnRNPA2B1 and *HIF-1α* mRNA. *HIF-1β* and *GLUT1* are used to negative controls. **b** Schematic of luciferase gene constructs flanked by untranslated regions of the HIF-1α transcript. **c** RNA-immunoprecipitation of hnRNPA2B1 with the untranslated region (UTR)-based luciferase constructs. Relative transcript levels of endogenous *HIF-1α* and exogenous luciferase genes were examined by RT-PCR. **d** UTR reporter assay was measured by luciferase enzyme activity in the cells transfected with each chimeric construct. Data presented as the mean ± SD of three experiments

To further investigate the mechanism of HIF-1α reduction by MO-460 treatment, we explored the interaction between hnRNPA2B1 and *HIF-1α* mRNA under in presence of MO-460. The hnRNPA2B1 protein and *HIF-1α* mRNA complex was immunoprecipitated from whole lysates of Hep3B cells cultured under mimetic hypoxia. But, this interaction was abolished in presence of MO-460. In addition, there was no detectable *HIF-1β* mRNA regardless of MO-460 treatment (Fig. 4a). This result suggests that hnRNPA2B1 binds to *HIF-1α* mRNA under mimetic hypoxia and that MO-460 inhibits their interaction.

And we further tested whether which region of *HIF-1α* mRNA is important for hnRNPA2B1 binding, Hep3B cells were transiently transfected with several luciferase-expression constructs possessing 5’-, 3’-, or both UTR regions of *HIF-1α* (Fig. 4b). An anti-hnRNPA2B1 antibody preferentially precipitated *luciferase* mRNA with the *HIF-1α* 3’-UTR (Fig. 4c). Similarly, luciferase activity was significantly reduced in hnRNPA2B1-depleted cells expressing luciferase constructs with the *HIF-1α* 3’-UTR (Fig. 4d). These results suggest that the 3’-UTR of the *HIF-1α* transcript is important for hnRNPA2B1 binding and regulation.

### hnRNPA2B1 is required for HIF-1α protein synthesis

**Fig. 5:**
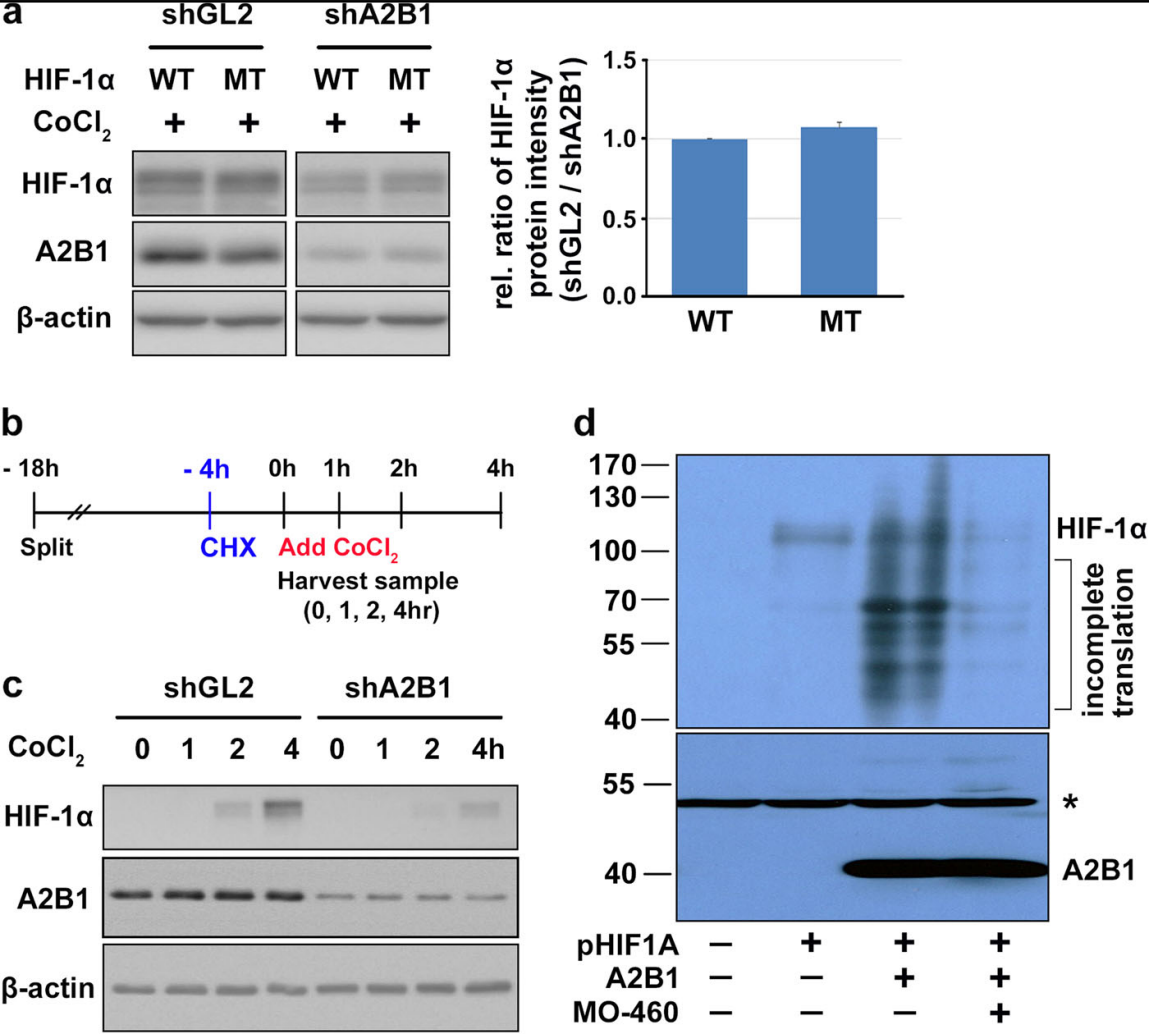
Inhibitory effect of MO-460 on HIF-1α protein levels occurs at the protein level. **a** Protein levels of hnRNPA2B1 (A2B1) and P402A/P564A mutant (MT) and wild-type HIF-1α in shGL2 (control) and shA2B1 stable cell lines under mimetic hypoxia were assessed by western blotting and the relative ratio of MT and WT HIF-1α in control and shA2B1 cells lines were quantified. **b** Schedule of newly synthesized HIF-1α protein experiments by CoCl_2_. Cycloheximide (CHX) was treated 4 h before adding CoCl_2_ for inhibiting de novo synthesis. **c** Western blotting of newly synthesized HIF-1α protein was performed in shGL or shhnRNPA2B1 stable cell lines. **d** In vitro transcription/translation assay of HIF-1α was performed in the presence or absence of hnRNPA2B1 protein and MO-460. Western blotting was performed with an anti-HIF-1α. Asterisk (*) indicates non-specific bands

HIF-1α undergoes hydroxylation at proline residues 402 and 564 and is ubiquitinated and subsequently targeted for proteasomal degradation under normoxic conditions^32^. To examine whether MO-460-mediated inhibition of HIF-1α accumulation was due to increased protein degradation, 2 missense mutations (P402A, P564A) were introduced into HIF-1α to prevent its ubiquitin-dependent degradation. Both wild-type and mutant HIF-1α proteins accumulated in the presence of hnRNPA2B1, but their levels were reduced in its absence under mimetic hypoxia (Fig. 5a). In the absence of hnRNPA2B1, the reduction was similar between wild-type and mutant HIF-1α, suggesting that protein degradation is not the primary mechanism of hnRNPA2B1 or MO-460-mediated regulation of HIF-1α protein levels.

Previous experiments investigating transcription suppression and protein degradation suggest that neither of these were the core pathway for the reduced accumulation of HIF-1α by MO-460 treatment or inhibition of its binding partner, hnRNPA2B1. Accumulation of HIF-1α, however, may be related to protein synthesis. We evaluated the effects of hnRNPA2B1 on HIF-1α protein synthesis under mimetic hypoxia. Cells were incubated with cycloheximide (CHX) for 4 h before addition of CoCl_2_ to prevent de novo protein synthesis, and then levels of newly synthesized protein were observed for up to 4 h (Fig. 5b). Under mimetic hypoxia, newly synthesized HIF-1α protein expression was diminished in the absence of hnRNPA2B1 (Fig. 5c). And, to verify the relationship between hnRNPA2B1 and MO-460 in HIF-1α protein translation, we performed an in vitro translation assay. Purified hnRNPA2B1 protein more enhanced HIF-1α translation than HIF-1α only, but MO-460 treatment attenuated this effect (Fig. 5d). These results strongly suggest that hnRNPA2B1 augments the translation of HIF-1α. In addition, the hnRNPA2B1-targeting small molecule, MO-460, can interfere with this process.

### MO-460 induces accumulation of stress granules (SGs) by regulating hnRNPA2B1

**Fig. 6:**
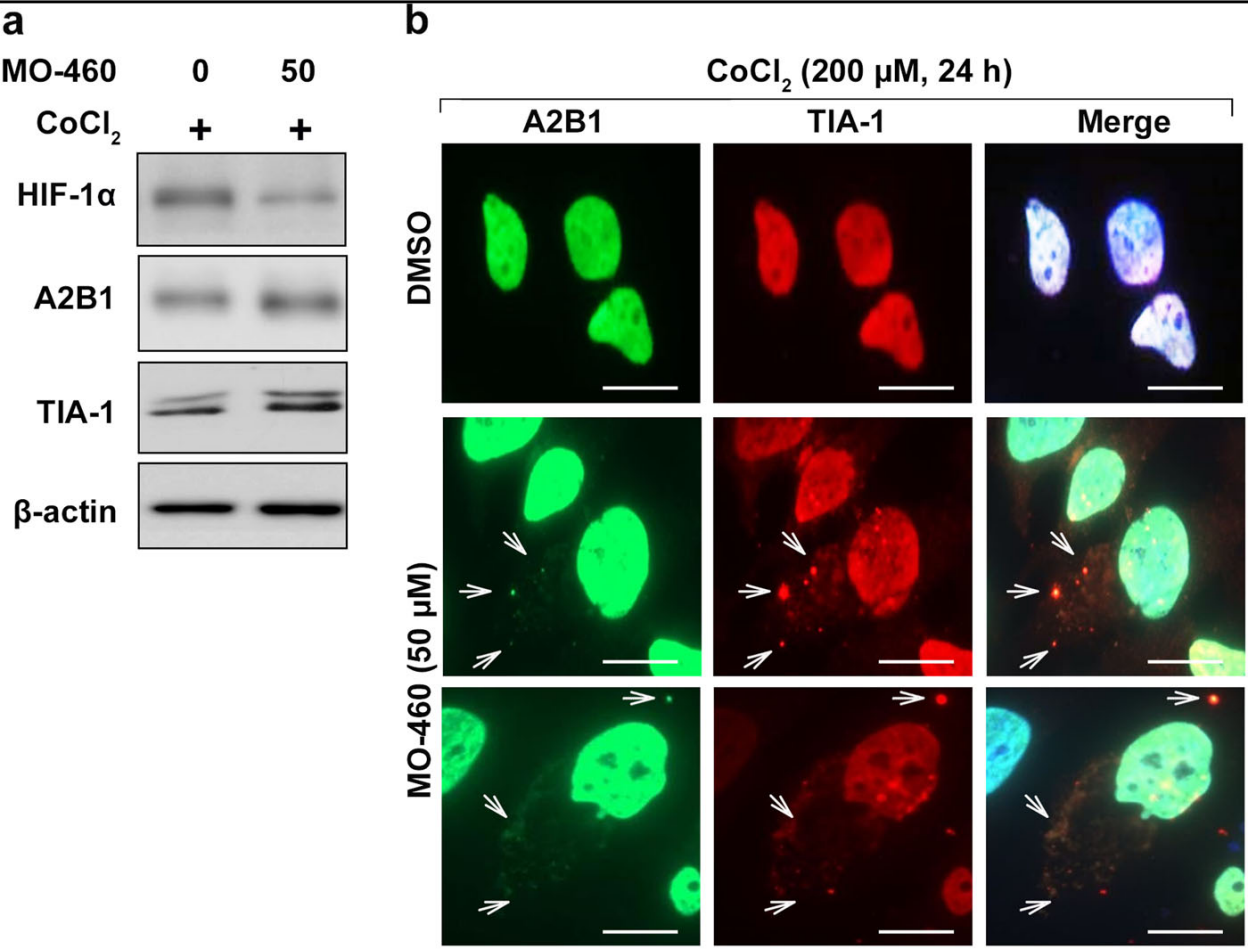
Inhibitory effect of MO-460 on HIF-1α protein translation is via stress granule formation. **a** Western blotting of indicated antibodies in Hep3B cells under mimetic hypoxia and the presence or absence of MO-460. **b** Immunostaining of cells grown under mimetic hypoxia described using anti-hnRNPA2B1, anti-TIA-1(red) and nuclei stain (blue). Scale bar, 20 μm

Both MO-460 treatment and hnRNPA2B1-inhibition appear to impede HIF-1α translation. In mammalian cells, cytotoxic stress triggers several signaling cascades that converge on stress granules, shuttling nuclear RNA-binding proteins such as T-cell intracellular antigen-1 (TIA-1) to the cytoplasm, and aggregating cellular mRNAs into TIA-1-containing complex, thus protein synthesis is greatly impaired^33^. To test whether hnRNPA2B1 was involved in stress granulation formation, sodium arsenite as a stress-inducing agent was treated with GFP and GFP-hnRNPA2B1 expressing cell lines. Unlike GFP, GFP-hnRNPA2B1 co-localized with TIA-1 (Supplementary Figure S8). And treatment with MO-460 causes formation of cytoplasmic dots in hnRNPA2B1 and it is co-localized with TIA-1, even the expression levels of hnRNPA2B1 and TIA-1 proteins were not altered  (Fig. 6a and b). These data imply that MO-460 relates to the regulation of HIF-1α protein synthesis by regulating hnRNPA2B1 via stress granules.

## Discussion

Cancer cells systematically change their transcriptome and subsequently their proteome in response to hypoxic challenges caused by rapid cell proliferation. These responses include adaptive mechanisms such as survival, division, motility, and cell differentiation^2,3^. Most of these transcriptional changes are orchestrated by the key regulatory transcription factor, HIF-1, which is also responsible for the resistance of cancer cells to chemotherapy^9,34,35^. HIF-1 is a heterodimer consisting of two HIF-1 subunits, alpha and beta. HIF-1α responds to and accumulates under hypoxic conditions, in contrast to constitutively expressed HIF-1β. Thus, HIF-1α has become an important therapeutic target for solid tumors. The roles of numerous small molecules used to treat various cancers by inhibiting HIF-1α activity have been characterized. However, additional compounds with verified mechanisms of action are expected to be developed as drugs for either single or combination therapies^32^.

Previous research has shown that the natural compound (R)-(-)-moracin-O suppress HIF-1α nuclear accumulation under hypoxic conditions, but the mechanism remained unclear^16^. To our knowledge, the current study is the first to identify and characterize the mechanism of HIF-1α suppression by MO-460. First, we identified that hnRNPA2B1 is a binding target of MO-460. hnRNPA2B1 is not only a biomarker for cancers, but also an oncogene that regulates tumor suppressors and other oncogenes in various cancers^27,36^. hnRNPA2B1 is overexpressed in cancer cell lines derived from many organs including the lung, breast, gastrointestinal tract, brain, cervix, and ovary^37–39^. Overexpression of hnRNPA2B1 is well-established as a biomarker for the early stages of non-small cell lung cancers, epithelial transformation, and carcinogenesis^40^. It also plays essential roles in carcinogenesis and the progression of cancer with respect to fundamental biological functions (e.g., glucose metabolism^41^, cell cycle^42^, cell proliferation^43^, invasive migration^18^). Specifically, its role in cell proliferation was supported by the observation that siRNA-mediated reduction of hnRNPA2B1 levels provoked rapid cell death by apoptosis in cell lines derived from diverse cancers of the cervix, colon, breast, ovary, and brain, but not in noncancerous fibroblastic or epithelial cell lines^38^. The results indicate that hnRNPA2B1 may provide a target for the treatment of a variety of cancers. Especially, the 28 amino acids in the C-terminal end of hnRNPA2B1 (GRD3) showed higher binding affinity than the full-length protein (Fig. 2 and Table 2). These binding affinity results suggest a strong physical interaction, which is a desirable property in potential drug candidates.

Second, we characterized the interaction between hnRNPA2B1 and 3’-UTR of *HIF-1α* mRNA and regulation of hnRNPA2B1 to HIF-1α. The hnRNPA2B1 protein belongs to a family of RNA-binding proteins with more than 20 members, which are abundantly expressed in most human tissues and play diverse roles in the post-transcriptional processing of mRNA and its subsequent packaging, transport, and translation^17^. According to previous studies, hnRNPA2B1 enhances cap-dependent translation without affecting internal ribosome entry site-dependent translation for transcripts containing RNA trafficking signals^19^. hnRNPA2, isoform of hnRNPA2B1 decreases the stability and translation of *GLUT1* by binding to the 3’-UTR under normoxia and de-represses translation by selectively inhibiting this interaction under hypoxic conditions^23^, and it acts as a regulator in the RNA splicing process and a natural compound, apigenin, inhibits its splicing activity^44^. However, the MO-460 target, hnRNPA2B1, has little effect in the case of *HIF-1α* RNA processing (Supplementary Figure S6).

Similar to other RNA-binding proteins, hnRNPA2B1 binds to *HIF-1α* transcripts (Fig. 4a) and has no direct interaction with the HIF-1α protein (Supplementary Figure S7). Moreover, hnRNPA2B1 binds specifically to *HIF-1α* mRNA, and not to other HIF-related transcripts such as *HIF-2α*, *HIF-3α*, and *HIF-1β*. Suppression of hnRNPA2B1 function by siRNA, shRNA, or MO-460 treatment resulted in the failure of HIF-1α protein accumulation under hypoxic conditions but had little effect (<10%) on HIF-1α transcript levels (Fig. 3a). This reduction is not sufficient to explain the large decrease in HIF-1α protein levels. In addition, even in normoxic conditions, MO-460 treatment did not affect *HIF-1α* transcript levels (Supplementary Figure S5a). These results suggest that hnRNPA2B1 has mainly impacts post-transcriptional or translational regulation rather than the transcription.

hnRNPA2B1 plays an important role in the accumulation of HIF-1α in Hep3B liver cancer cells under mimetic hypoxia. Our RNA immunoprecipitation experiments showed that hnRNPA2B1 preferentially binds the 3’-UTR of *HIF-1α* mRNA, although the quantity of luciferase transcripts expressed from each construct was similar (Fig. 4c), probably due to the strong CMV promoter activity (Fig. 4b). The luciferase transcription levels from each construct were similar regardless of hnRNPA2B1 depletion in the cells in the absence of the *HIF-1α* 3’-UTR. In the presence of the *HIF-1α* 3’-UTR, however, the luciferase activity decreased by 20–40% when hnRNPA2B1 was knocked down (Fig. 4d). The results obtained from the RNA binding and UTR reporter assays suggest that hnRNPA2B1 binds to the 3’-UTR of *HIF-1α* mRNA, enhancing HIF-1α protein expression.

Third, accumulation of HIF-1α protein in cells under hypoxic conditions may be related to an inhibition in degradation or an increase in protein synthesis^2^. Under normoxia, HIF-1α protein is rapidly ubiquitinated by the von Hippel Lindau protein (pVHL) complex and subsequently targeted for proteasomal degradation^45^. MO-460 treatment under hypoxic conditions completely eliminated the accumulation of HIF-1α (Fig. 1 and Supplementary Figure S3). Wild-type and degradation-defective (MT) HIF-1α exhibited little difference in nuclear accumulation or degradation under mimetic hypoxia in the presence or absence of hnRNPA2B1 (Fig. 5a). We excluded the possibility that the lack of HIF-1α accumulation after MO-460 treatment or hnRNPA2B1 knockdown was due to increased protein degradation. Finally, we found that treatment with MO-460 or hnRNPA2B1 depletion under hypoxic conditions decreased HIF-1α protein translation (Fig. 5).

Then, we investigated how protein synthesis is affected by MO-460 treatment or hnRNPA2B1 inhibition. A recent study reported that a mutation in hnRNPA2B1 promotes the formation of stress granules associated with RNAs^46,47^. The formation of stress granules is an inhibitory process for protein synthesis^48,49^. Here, we observed that hnRNPA2B1 colocalized with TIA-1, a stress granule marker, with sodium arsenite treatment, a known stress granule inducer (Supplementary Figure S8). Thus, we confirmed stress granule formation in response to MO-460 treatment (Fig. 6). These results suggest that MO-460 treatment might be caused inhibition of HIF-1α protein synthesis via stress granules mediated by hnRNPA2B1.

Although it is premature to discuss the extent of MO-460 targets, it is possible that the functions of the 11 selected MO-460 binding proteins (Supplementary Figure S2) could also be affected by MO-460 binding. Functional inhibition of any of these proteins would exert a considerable effect on carcinogenesis, angiogenesis, and metastasis considering the roles of nucleolin, for example, in angiogenesis and tumor development^50,51^ and of nucleophosmin in the ARF-p53 tumor suppressor pathway^52^. However, from our results, among MO-460 targets, hnRNPA2B1 seems to be valuable as an anti-cancer target on HIF-1α inhibition. Functional suppression of hnRNPA2B1 inhibits alternative splicing of pyruvate kinase muscle isozyme M2 (PKM2), a key enzyme for glucose metabolism in tumors^41^, and tumor protein P53 inducible nuclear protein2 (TP53INP2), an important component of invasive cell movement^18^. Moreover, (R)-(-)-moracin-O and its synthetic derivative, MO-460, bind to hnRNPA2B1 (Figs. 1 and 2) and suppress HIF-1α nuclear accumulation though inhibition of protein synthesis under hypoxic conditions. This benzofuran-based small molecule, MO-460, warrants further exploration as a drug candidate for the development of novel anticancer therapeutics.

## Supplementary information

Supplementary Figure Legends

Supplementary Materials and Methods

Supplementary Table S1

Supplementary Figure S1

Supplementary Figure S2

Supplementary Figure S3

Supplementary Figure S4

Supplementary Figure S5

Supplementary Figure S6

Supplementary Figure S7

Supplementary Figure S8
