# Supplementary material for: Mechanism of the natural product moracin-O derived MO-460 and its targeting protein hnRNPA2B1 on HIF-1α inhibition
Source: Exp Mol Med. 2019 Feb 12;51(2):10. doi: 10.1038/s12276-018-0200-4 (PMC6372683; doi:10.1038/s12276-018-0200-4)
Supplement: Supplementary file 1 — Supplementary Figure Legends [file 12276_2018_200_MOESM1_ESM.docx]

**Supplementary Figure legends**

**Supplementary Figure S1.** (**a**) Diagram of protein affinity-capture method based on binding to biotin-MO-460. (**b**) Pull-down assay results of Biotin-MO-460 with streptavidin beads in nuclear and cytosol fractions. The top 12 proteins identified as candidates by mass spectroscopy are indicated with arrows.

**Supplementary Figure S2.** HIF-1α staining after knockdown of MO-460-binding candidates in cytosolic fractions under mimetic hypoxia. (**a**) Knockdown in each batch of cells was confirmed by western blotting with the indicated antibodies. Equal protein loading was confirmed with Coomassie brilliant blue (CBB) staining. (**b**) Immunostaining was performed with an anti-HIF-1α antibody. (**c**) Western blotting of anti-HIF-1α, anti-HSP90β, and anti-A2B1 under treatment of indicated siRNAs. Asterisk (*) in part (b) indicates that cell death was evident. Scale bar, 20 μm. (**d**) Diagram of shRNA-targeted location mapped on human hnRNPA2B1 mRNA, and western blotting of hnRNPA2-targeting lentiviral constructs.

**Supplementary Figure S3.** HIF-1α inhibition following treatment with MO-460 or knockdown of hnRNPA2B1 under hypoxic condition for 24 h. (O_2_ 1%) Western blotting and immunostaining of HIF-1α under (**a**-**b**) treatment of MO-460 or (**c**-**d**) knockdown of hnRNPA2B1. Scale bar, 20 μm.

**Supplementary Figure S4.** Purification of His-tagged hnRNPA2B1, its domain protein, and various GST-fused GRD constructs. (**a**) Purification of full-length His-tagged hnRNPA2B1 and its domains, and (**b**) purified GST-fused GRD protein shown using CBB-stained gel. Asterisk (*) indicates non-specific bands. RRM, RNA recognition motif; GRD, glycine-rich domain.

**Supplementary Figure S5.** RT-PCR analysis of HIF-1α. Hep3B cells were treated with MO-460 at the indicated concentrations in the (**a**) absence or (**b**) presence of CoCl_2_ for 24 h. The relative levels of *HIF-1α* mRNA were quantified and normalized to those of *GAPDH*. ns, no significant difference.

**Supplementary Figure S6.** Alternative splicing of HIF-1α. (**a**) Pre-mRNA schematic representation of HIF-1α. HIF-1α splicing primers were designed to amplify PCR products from the end of each exon to the start of the next intron. (**b**) Schematic representation of the amplification of HIF-1α pre-mRNA and mRNA bands with specific primers. (**c**) RT-PCR analysis with HIF-1α splicing primers. Template cDNAs were used for the lentiviral-infected control (shGL2) and knockdown of hnRNPA2B1 (shA2B1). Genomic DNA obtained from CCL2 cells was used for the positive control.

**Supplementary Figure S7.** Interaction between hnRNPA2B1 protein and *HIF-1α* mRNA. (**a**) Hep3B cell lysates and (**b**) lysates from HEK293T cells transfected with either GFP vector or GFP-hnRNPA2B1 were RNA-immunoprecipitated (RNA-IP) with anti-hnRNPA2B1 or anti-GFP and subjected to western blotting (upper panels) or RT-PCR (lower panels), as indicated.

**Supplementary Figure S8.** hnRNPA2B1 protein colocalizes with stress granules under sodium arsenite induction conditions. HEK293T cells transfected with either GFP vector or GFP-hnRNPA2B1 were treated with 0.5 mM sodium arsenite for 30 min, then the cells were stained with anti-TIA-1. Arrows show stress granules. Asterisks (*) indicate the cells that were not transduced with the lentivirus. Scale bar, 20 μm.
