# Supplementary material for: Mechanism of the natural product moracin-O derived MO-460 and its targeting protein hnRNPA2B1 on HIF-1α inhibition
Source: Exp Mol Med. 2019 Feb 12;51(2):10. doi: 10.1038/s12276-018-0200-4 (PMC6372683; doi:10.1038/s12276-018-0200-4)
Supplement: Supplementary file 2 — Supplementary Materials and Methods [file 12276_2018_200_MOESM2_ESM.docx]

**Supplementary Materials and Methods.**

**Supplementary Materials and methods 1.** Synthesis of MO-460 and its biotin conjugated chemical probes (Biotin-MO-460)

**Scheme 1: Synthesis of MO-460 and its chemical probes**

**
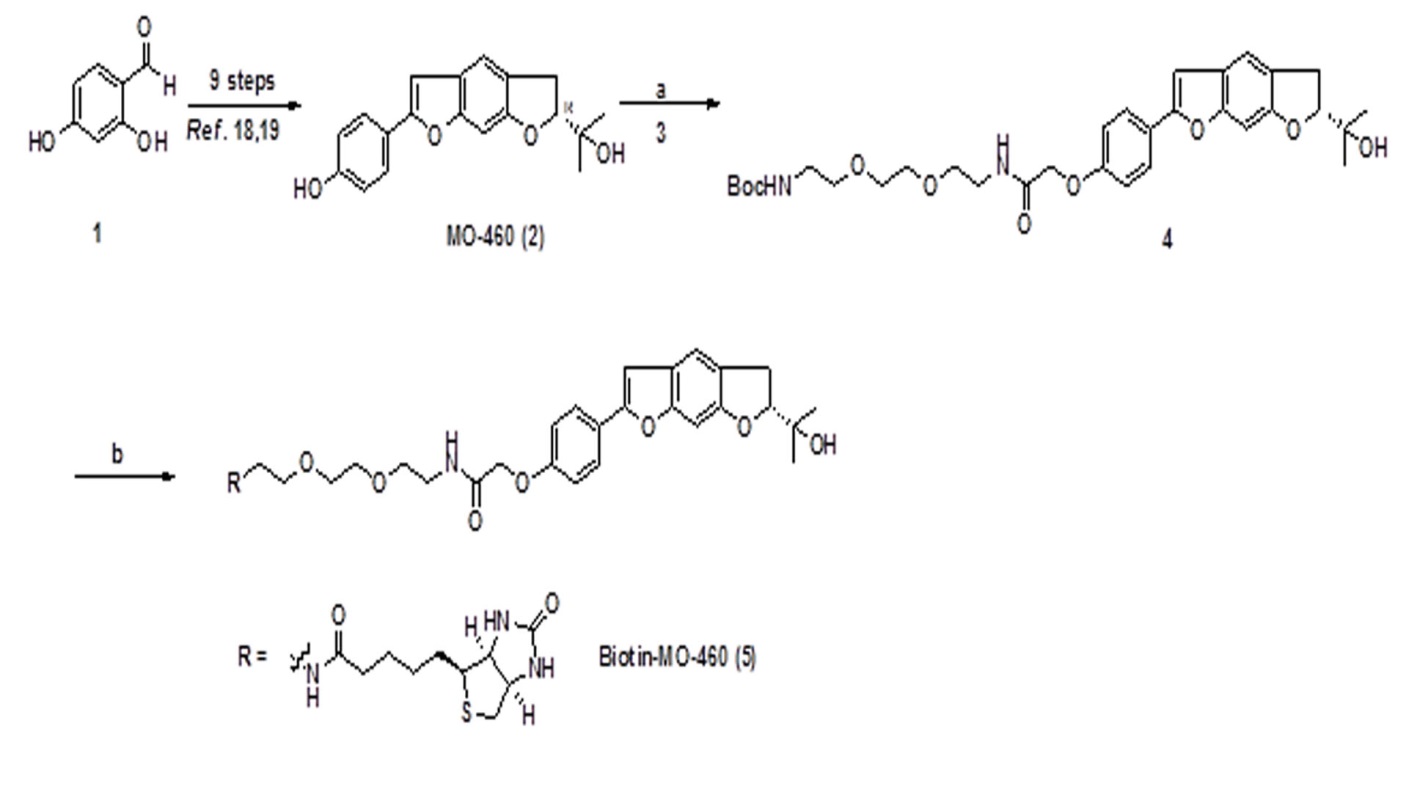
**

**^a^Reagents and Conditions:** a) K_2_CO_3_, Cs_2_CO_3_, KI, acetone/DMF, (2-{2-[2-(2-chloro-acetylamino)-ethoxy]-ethoxy}-ethyl)-carbamic acid tert-butyl ester (**3**); b) (i) TFA, MC; (ii) TEA, (+)**-**Biotin *N*-hydroxysuccinimide ester, DMF for Biotin-MO-460 (**5**)

Synthesis of Biotin-MO-460 carrying structural units like the biotin reporter group was described in Scheme1. MO-460 (**2)** was synthesized starting from commercially available 2,4-dihydroxy benzaldehyde **1** by our previously reported method. Next, **2** was directly treated with (2-{2-[2-(2-chloro-acetylamino)-ethoxy]-ethoxy}-ethyl)-carbamic acid *tert*-butyl ester **3** under basic conditions, to yield derivative with linker **4**. This derivative was further subjected for Boc deprotection followed by reaction with (+)**-**biotin *N*-hydroxysuccinimide ester or fluorescein isothiocyanate to furnish the corresponding Biotin-MO-460 (**5**) probes, respectively.

**EXPERIMENTAL SECTION:**

All the commercial chemicals were of reagent grade and were used without further purification. Solvents were dried with standard procedures. All the reactions were carried out under an atmosphere of dried argon in flame-dried glassware. The proton nuclear magnetic resonance (^1^H-NMR) spectra were determined on a Varian (300 MHz, 400 MHz or 500 MHz) spectrometer (Varian Medical Systems, Inc., Palo Alto, CA, USA). ^13^C-NMR spectra were recorded on a Varian (100 or 125 MHz) spectrometer. The chemical shifts are provided in parts per million (ppm) downfield with coupling constants in hertz (Hz). The mass spectra were recorded using high-resolution mass spectrometry (HRMS) (electron ionization MS) obtained on a JMS-700 mass spectrometer (Jeol, Japan) or using HRMS (electrospray ionization MS) obtained on a G2 QTOF mass spectrometer. The products from all the reactions were purified by flash column chromatography using silica gel 60 (230–400 mesh Kieselgel 60). Additionally, thin-layer chromatography on 0.25-mm silica plates (E. Merck; silica gel 60 F254) was used to monitor reactions. The purity of the final products was checked by reversed phase high-pressure liquid chromatography (RP*-*HPLC), which was performed on a Waters Corp. HPLC system equipped with an ultraviolet (UV) detector set at 254 nm. The mobile phases used were (A) H_2_O containing 0.05% trifluoroacetic acid, and (B) CH_3_CN. HPLC employed a YMC Hydrosphere C_18_ (HS-302) column (5-µm particle size, 12-nm pore size) that was 4.6 mm in diameter × 150 mm in size with a flow rate of 1.0 mL/min. The compound purity was assessed either using (Method A) a gradient of 20% B to 100% B in 35 min or (Method B) a gradient of 25% B to 100% B in 35 min. All biologically evaluated compounds purity were >95% in both method A and method B.

**(R)-4-[6-(1-Hydroxy-1-methylethyl)-5,6-dihydro-benzo[1,2-b;5,4-b’]difuran-2-yl]-phenol (2):** Synthetic procedure was followed as per our previous method.^7^

Obtained as a white solid (0.1 g, 85.0 % yield). Melting Point: 227.6-230.6 ˚C (dec); ^1^H NMR (CD_3_OD, 400 MHz) *δ* 7.62 (d, *J* = 8.4 Hz, 2H), 7.26 (s, 1H), 6.82 (d, *J* = 8.7 Hz, 2H), 6.81 (s, 1H), 6.80 (s, 1H), 4.63 (t, *J* = 8.4 Hz, 1H), 3.22 (dd, *J* = 2.4 Hz, 8.7 Hz, 2H), 1.27 (s, 3H), 1.24 (s, 3H); ^13^C NMR (CD_3_OD, 100 MHz): *δ* 159.4, 158.8, 156.7, 156.3, 126.8, 124.8, 124.4, 124.0, 116.7, 116.6, 100.1, 93.3, 91.4, 72.6, 31.3, 30.7, 25.4, 25.3; MS (EI) *m/z* 310 (M^+^); HRMS (EI) *m/z* calcd. C_19_H_18_O_4_ [M^+^] 310.1205, found: 310.1208.; Purity = 100 % (as determined by RP-HPLC, Method A, *t*_R_ = 13.775 min; Method B, *t*_R_ = 12.277 min); Specific Optical Rotation: [α]^25^_D_ = -36 (*c* = 0.1, MeOH).

**(2-{2-[2-(2-Chloro-acetylamino)-ethoxy]-ethoxy}-ethyl)-carbamic acid *tert*-butyl ester (3):** 1,2-Bis(2-aminoethoxy)ethane (300.0 mg, 2.03 mmol) was dissolved in a DCM (5 mL) and added triethylamine (TEA) (0.3 mL) followed by solution of di-*tert*-butyl dicarbonate (133.2 mg, 0.61 mmol) in DCM (4 mL) was added dropwise to this mixture with vigorous stirring. The mixture was stirred overnight at room temperature. The reaction mixture was washed with a solution of sodium chloride. The combined organic extracts were dried on anhydrous MgSO_4_, filtered and the solvent was removed with rotary evaporator to give monoprotected diamine as yellow oil (101.0 mg, 66.7 % yield). TLC control showed almost no bis-protected diamine. The yellow oil was used without further purification. A solution of chloroacetyl chloride (178.0 mg, 1.58 mmol) in CH_2_Cl_2_ (3 mL) was added dropwise over 20 min to a solution of monoprotected diamine (327.5 mg, 1.32 mmol) and TEA in CH_2_Cl_2_ (3 mL) at -20 ˚C. The resulting brown solution was left to stir at room temperature for 24 h. The reaction solution was removed in *vacuo*, and the obtained residue was dissolved in CH_2_Cl_2_ and washed with a solution of sodium carbonate. The combined organic extracts were dried on anhydrous MgSO_4_, filtered and concentrated with rotary evaporator to give **3** as brown oil (0.302 g, 70.4 % yield). ^1^H NMR (CDCl_3_, 300 MHz): *δ* 7.03 (s, 1H), 5.06 (s, 1H), 3.95 (s, 2H), 3.38 – 3.51 (m, 10H), 3.21-3.20 (m, 2H), 1.33 (s, 9H).

**(R)-(2-{2-[2-(2-{4-[6-(1-Hydroxy-1-methyl-ethyl)-5,6-dihydro-benzo[1,2-b;5,4-b']difuran-2-****yl]-phenoxy}-acetylamino)-ethoxy]-ethoxy}-ethyl)-carbamic acid tert-butyl ester** **(4):** To the solution of **3** (47 mg, 0.15 mmol) in anhydrous acetone (6 mL) was subsequently added compound **2** (98 mg, 0.30 mmol), Cs_2_CO_3_ (98.7 mg, 0.30 mmol) and KI (25 mg, 0.15 mmol). The reaction mixture was heated at 60 ˚C for 12 h and cooled to room temperature. The mixture was evaporated under reduced pressure and the residue was washed with water. The solution was extracted with ethyl acetate and the combined organic layers were dried over anhydrous MgSO_4_, filtered and concentrated in *vacuo*. The crude product was purified by preparative TLC (n-Hexane:EtOAc = 2:1) to obtain **4** as white solid (0.03 g, 32.9 % yield); ^1^H NMR (CD_3_OD, 300 MHz): *δ* 7.75 (d, *J* = 9.0 Hz, 2H), 7.28 (s, 1H), 7.06 (d, *J* = 9.3 Hz, 2H), 6.92 (s, 1H), 6.86 (s, 1H), 4.63 (m, 1H), 4.57 (s, 2H), 3.60-3.46 (m, 10H), 3.26-3.20 (m, 4H), 1.41 (s, 9H), 1.27 (s, 3H), 1.24 (s, 3H); MS (EI) *m/z* 598 (M^+^).

**(R)-5-(2-Oxo-hexahydro-thieno[3,4-d]-imidazol-4-yl)-pentanoic acid-(2-{2-[2-(2-{4-[6-(1-hydroxy-1-methyl-ethyl)-5,6-dihydro-benzo[1,2-b;5,4-b']difuran-2-yl]-phenoxy}-acetylamino)-ethoxy]-ethoxy}-ethyl)-amide (5):**  The compound **4** (30 mg, 0.05 mmol) was dissolved in the 3 mL mixture of TFA and CH_2_Cl_2_ (1:3) and the solution was stirred at room temperature for 1 h. Then the solvents were removed and co-evaporated with toluene for 3 times to obtain crude free amine product. The amine product without further purification was dissolved in the DMF and added TEA. After that, biotin (20 mg, 0.06 mmol) was added to the solution and stirred at room temperature overnight. The reaction was quenched by water and extracted with ethyl acetate. The organic layers were dried over anhydrous MgSO_4_ and filtered, concentrated under reduced pressure. The crude product was purified by Prep-TLC (CH_2_Cl_2_:MeOH = 20:1) to give compound **5** as a white solid (24 mg, 67 % yield); Melting Point = 165.1-168.2 °C (dec); ^1^H NMR (CD_3_OD, 400 MHz): *δ* 7.75 (d, *J* = 12.0 Hz, 2H), 7.29 (s, 1H), 7.06 (d, *J* = 12.0 Hz, 2H), 6.92 (s, 1H), 6.87 (s, 1H), 4.64 (t, *J* = 12.0 Hz, 1H), 4.57 (s, 1H), 4.43-4.40 (m, 1H), 4.23-4.22 (m, 1H), 3.60-3.47 (m, 10H), 3.34-3.33 (m, 2H), 3.24-3.20 (m, 2H), 3.12-3.10 (m, 1H), 2.88-2.83 (m, 1H), 2.66 (d, *J* = 12.0 Hz, 1H), 2.16 (t, *J* = 12.0 Hz, 2H), 1.66-1.52 (m, 4H), 1.39-1.35 (m, 2H), 1.27 (s, 3H), 1.24 (s, 3H); ^13^C NMR (CD_3_OD, 100 MHz): *δ* 176.2, 171.1, 159.8, 159.0, 156.4, 155.8, 126.8, 126.3, 125.2, 124.3, 117.0, 116.4, 101.4, 93.4, 91.4, 72.6, 71.4, 71.3, 70.7, 70.5, 68.4, 63.4, 61.6, 57.0, 41.1, 40.3, 40.0, 36.8, 31.2, 29.8, 29.5, 26.9, 25.4, 25.3; MS (FAB^+^) *m/z* 725 (M+H^+^); HRMS (FAB^+^) *m/z* calcd. C_37_H_49_N_4_O_9_S [M+H^+^] 725.3220, found: 725.3220; Purity = 100 % (as determined by RP-HPLC, Method A, *t*_R_ = 12.16 min; Method B, *t*_R_ = 10.59 min). Specific Optical Rotation: [α]^25^_D_ = -6 (*c* = 0.1, MeOH).

**(R)-4-[6-(1-Hydroxy-1-methylethyl)-5,6-dihydro-benzo[1,2-b;5,4-b’]difuran-2-yl]-phenol (2)**


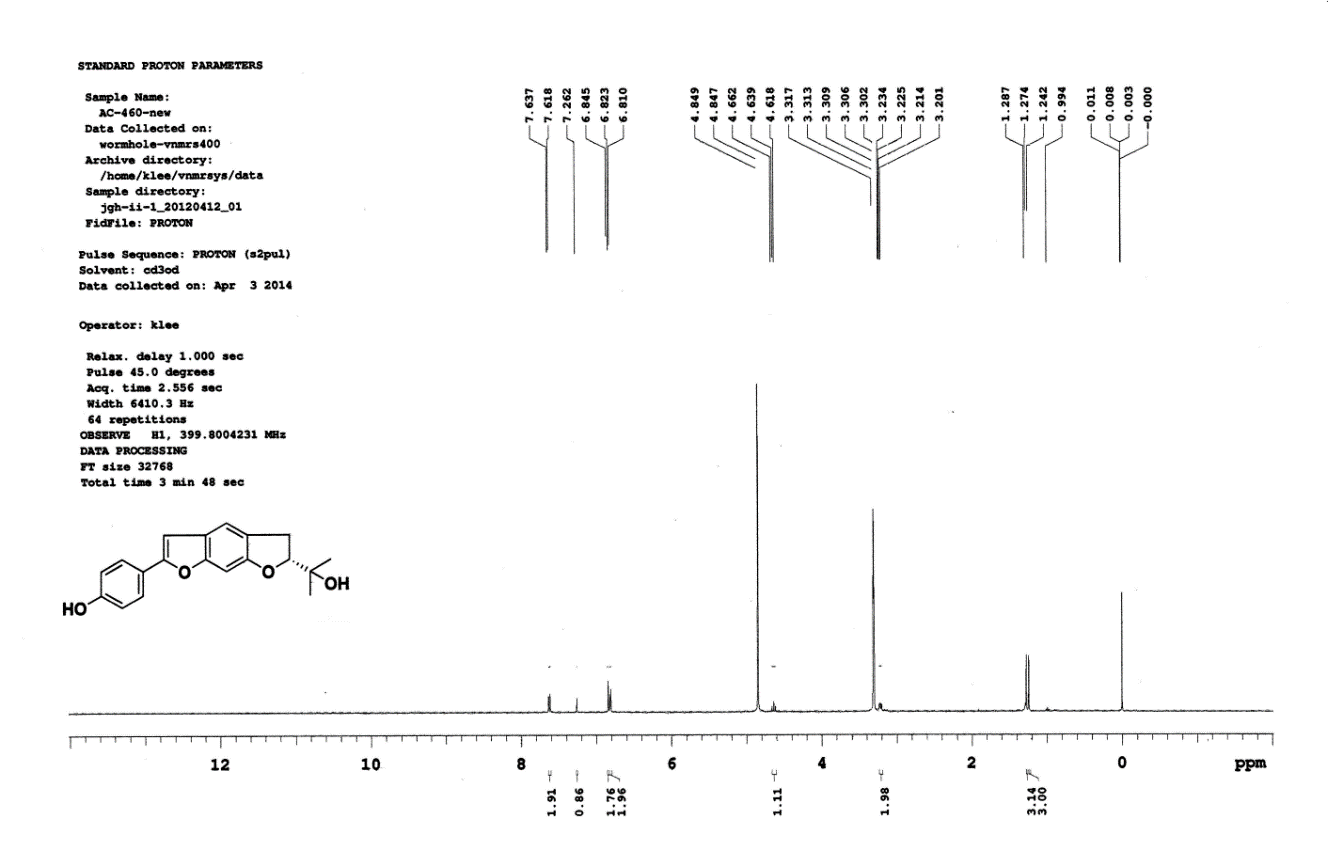


^1^H-NMR (CD_3_OD, 400 MHz)


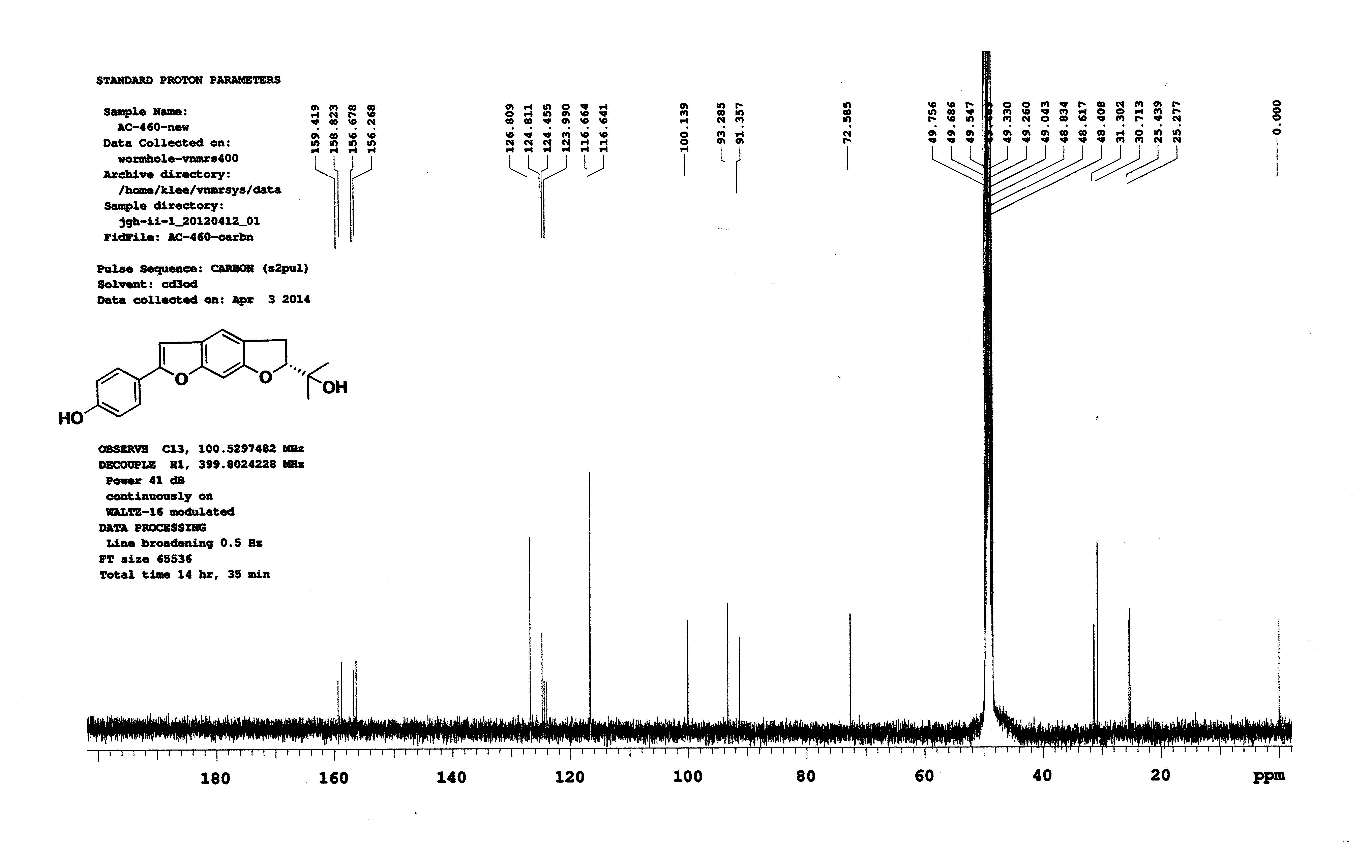


^13^C-NMR (CD_3_OD, 100 MHz)

**(*R*)-5-(2-Oxo-hexahydro-thieno[3,4-d]-imidazol-4-yl)-pentanoic acid-(2-{2-[2-(2-{4-[6-(1-hydroxy-1-methyl-ethyl)-5,6-dihydro-benzo[1,2-b;5,4-b']difuran-2-yl]-phenoxy}-acetyl-amino)-ethoxy]-ethoxy}-ethyl)-amide (5)**

^1^H-NMR (CD_3_OD, 400 MHz)
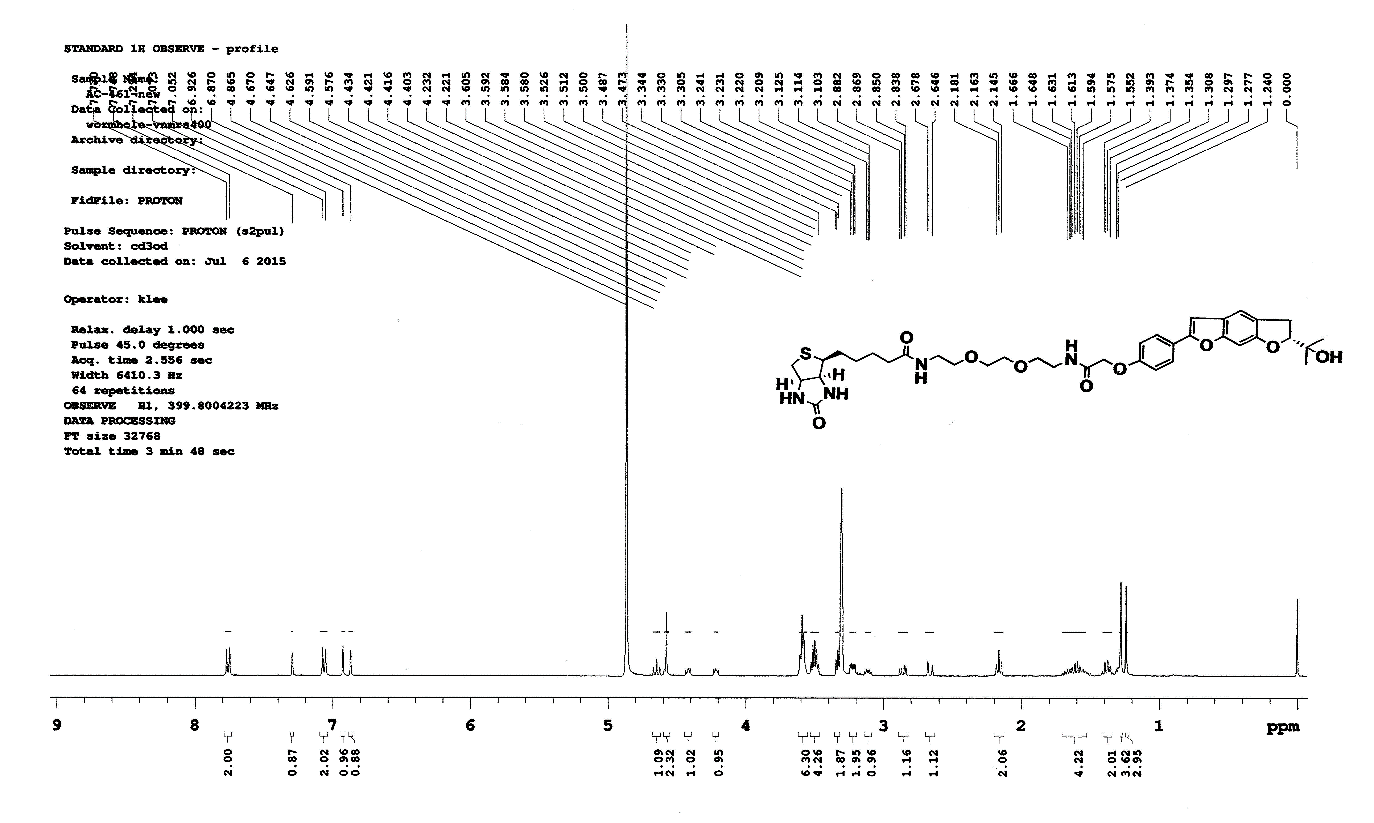


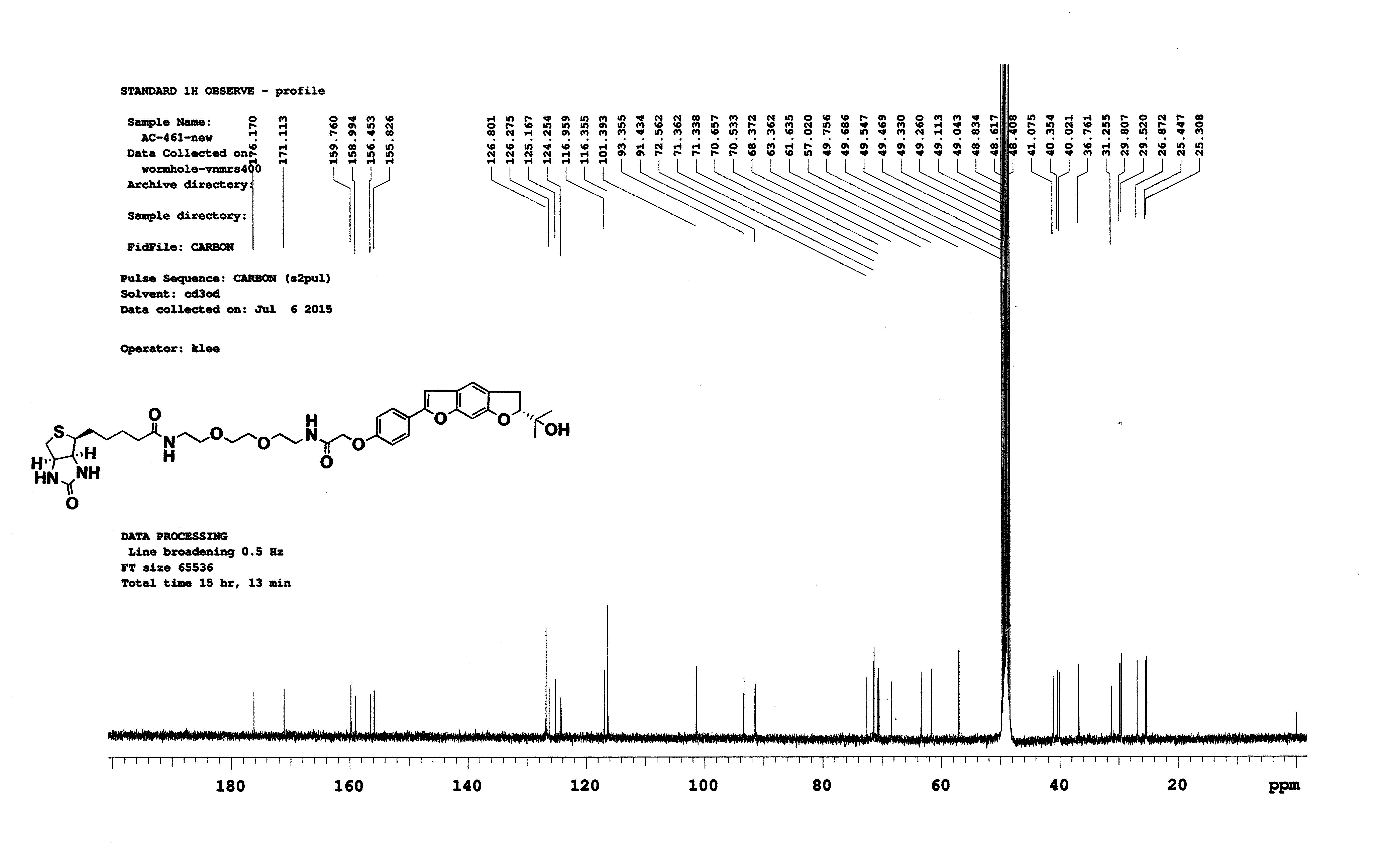


^13^C-NMR (CD_3_OD, 100 MHz)

**Supplementary Materials and Methods 2.** List of antibodies used in this study.

|  | Protein | Antibody |
| --- | --- | --- |
| 1 | NonO | Abcam, ab45359 |
| 2 | hnRNPA2B1 | SCBT, sc-53531 |
|  |  | Novus, NB-120-6102 |
|  |  | generated for this study |
| 3 | hnRNPA1 | SCBT, sc-166511 |
| 4 | hnRNPH1 | SCBT, sc-15387 |
| 5 | Aldolase A | ACRIS-AB, AP12349PU-N |
| 6 | HSP90β | SCBT, sc-1057 |
| 7 | FUBP1 | SCBT, sc-374342 |
| 8 | nucleolin | SCBT, sc-8031 |
| 9 | VCP | SCBT, sc-20799 |
| 10 | NPM3 | SCBT, sc-5564 |
| 11 | DDX17 | Abcam, ab24601 |
| 12 | GAPDH | SCBT, sc-35575 |
| 13 | HIF-1α | SCT, 3716 |
|  |  | Abcam, ab8366 |
| 14 | GFP | SCBT, sc-8334 |
| 15 |  | MBL, D-153-9 |
| 16 | TIA-1 | SCBT, sc-1751 |
| 17 | β-actin | Sigma, A5316 |

**Supplementary Materials and Methods 3.** List of plasmids used in this study for ectopic expression and depletion of targeted proteins.

| Gene | Vector | Insert | Enzymes of References | |
| --- | --- | --- | --- | --- |
|  |  |  | Vector | Insert |
| hnRNPA2B1 | pCDNA HA vector | hnRNPB1 | Origene Tech. Inc.  (Cat. Sc313092) | |
|  | pShuttle EGFP vector | hnRNPA2B1(1-353aa) | KpnI/NotI | KpnI/NotI |
|  | pShuttle EGFP vector | hnRNPA2B1(1-110aa) | KpnI/NotI | KpnI/NotI |
|  | pShuttle EGFP vector | hnRNPA2B1 (111-200aa) | KpnI/NotI | KpnI/NotI |
|  | pShuttle EGFP vector | hnRNPA2B1 (201-353aa) | KpnI/NotI | KpnI/NotI |
|  | pHR’CMV SV40 Puro | EGFP::hnRNPA2B1  (1-353aa) | EcoRI::ⓑ | AgeI/NotI::ⓑ |
|  | pHR’CMV SV40 Puro | EGFP::hnRNPA2B1  (1-110aa) | EcoRI::ⓑ | AgeI/NotI::ⓑ |
|  | pHR’CMV SV40 Puro | EGFP::hnRNPA2B1  (111-200aa) | EcoRI::ⓑ | AgeI/NotI::ⓑ |
|  | pHR’CMV SV40 Puro | EGFP::hnRNPA2B1  (201-353aa) | EcoRI::ⓑ | AgeI/NotI::ⓑ |
|  | pET28A vector | hnRNPA2B1 (1-353aa) | BamHI/NotI | BglII/NotI |
|  | pET28A vector | hnRNPA2B1(1-200aa) | BamHI/NotI | BglII/NotI |
|  | pET28A vector | hnRNPA2B1(201-353aa) | BamHI/NotI | BglII/NotI |
|  | pGEX6P-1 vector | hnRNPA2B1(201-250aa) | BamHI/NotI | BamHI/NotI |
|  | pGEX6P-1 vector | hnRNPA2B1(251-300aa) | BamHI/NotI | BamHI/NotI |
|  | pGEX6P-1 vector | hnRNPA2B1(301-353aa) | BamHI/NotI | BamHI/NotI |
|  | pGEX6P-1 vector | hnRNPA2B1(226-275aa) | BamHI/NotI | BamHI/NotI |
|  | pGEX6P-1 vector | hnRNPA2B1(276-325aa) | BamHI/NotI | BamHI/NotI |
| *HIF-1α UTR* Luc. assay | pCDNA3.1 vector | Luciferase(Luc) |  |  |
|  | pCDNA3.1 vector | 5’UTR-Luc-3’UTR |  |  |
|  | pCDNA3.1 vector | 5’UTR-Luc |  |  |
|  | pCDNA3.1 vector | Luc-3’UTR |  |  |
|  | pBabe-Puro HA | HIF-1*α* WT | Addgene  (Yan et al., 2007) | |
|  | pBabe-Puro HA | HIF-1*α* P402/564A |  |  |
| shRNA | pLKO.1 puromycin | sh Con. (shGL2) | (Soung *et al*., 2009) | |
|  | pLKO.1 puromycin | shhnRNPA2B1(shA2B1)#1 | AgeI/EcoRI | AgeI/EcoRI |
|  | pLKO.1 puromycin | shhnRNPA2B1(shA2B1)#3 | AgeI/EcoRI | AgeI/EcoRI |
| *in vitro* translation | pOTB7 | Human HIF1A  (Accession :NM001530) | KHGB  (Daejeon, Rep of Korea) | |

ⓑ : After digestion, DNAs were incubated with Klenow fragment and dNTP for 15 min at room temperature to make blunt ends

**Supplementary Materials and Methods 4.** List of RNAi used in this study and their sequences.

|  | Protein | RANi Cat.No | Sequence |
| --- | --- | --- | --- |
| siRNA | NonO | SCBT, sc-38163 |  |
|  | hnRNPA2B1 | SCBT, sc-53531 |  |
|  | hnRNPA1 | SCBT, sc-35575 |  |
|  | hnRNPH1 | Origene, sr302170 |  |
|  | Aldolase A | SCBT, sc-29664 |  |
|  | HSP90β | SCBT, sc-35606 |  |
|  | FUBP1 | Origene, sr305867 |  |
|  | nucleolin | SCBT, sc-29230 |  |
|  | VCP | SCBT, sc-37187 |  |
|  | NPM3 | SCBT, sc-29771 |  |
|  | DDX17 | Origene, sr307149 |  |
|  | GAPDH | SCBT, sc-35448 |  |
|  | Control | SCBT, sc-37007 |  |
| shRNA | Control (for GL2) |  | CGUACGCGGAAUACUUCGA |
|  | hnRNPA2B1 #1 |  | GGAUCAUGGUGUAAUAAGAUU (55) |
|  | hnRNPA2B1 #2 |  | GGAUGGCUAUAAUGGGUAU (723) |
|  | hnRNPA2B1 #3 |  | CAGUUCCGUAAGCUCUUUAUU (1467) |

**Supplementary Materials and Methods 5.** List of primer sets for this study

|  | **Gene** | **Sequence** | **Information** | **Exp.** |
| --- | --- | --- | --- | --- |
| 1 | *HIF-1α* | CTCACAGATGATGGTACATG | exon4_81F | RIP |
|  |  | AATCAAACACACTGTGTCCAG | exon5_177R | RIP |
|  |  | TGACCAGTTATGATTGTGAAGT | 2788F | RT |
|  |  | GTAATGAGCCACCAGTGT | 2933R | RT |
| 2 | *HIF-2α* | CTCTCCAACAAGCTGAAGCT | exon13_59F | RIP |
|  |  | AATGTGAGGTGCTGCCACC | exon14_31R | RIP |
| 3 | *HIF-3α* | CCTGTGACCAAGAGGAGCT | exon4_41F | RIP |
|  |  | GGAGAAGCACCGCTCCGT | exon5_47R | RIP |
| 4 | *HIF-1β* | GGCAGTAGCTCTGTGGACC | exon9_5F | RIP |
|  |  | CAGGCCTTGATGTAGCCTG | exon10_75R | RIP |
| 5 | *GLUT1* | AGGAGTTCTACAACCAGACAT | exon3_8F | RIP |
|  |  | CGGACACGAAGGCCAGCA | exon4_44R | RIP |
| 6 | *hnRNP A2B1* | AAGTTGTAGGTTGGCTGTT | 2971F | RT |
|  |  | CTTCAGTGGTGGTCTTAGG | 3113R | RT |
|  |  | ACGGAGGTGGTTATGACA | 1064F | RT |
|  |  | GTAGTTAGAAGGTTGCTGGTT | 1155R | RT |
| 7 | *GAPDH* | TGTTGCCATCAATGACCCCT | 312F | RT |
|  |  | CTCCACGACGTACTCAGCG | 513R | RT |
| 8 | *Luciferase* | AGAGATACGCCCTGGTTCCT | 92F | RT |
|  |  | ATAAATAACGCGCCCAACAC | 293R | RT |

RIP: RNA immune-precipitation, RT: Reverse Transcription.

**Supplementary Materials and Methods 6.** List of primer sets for HIF1A splicing observation.

| **HIF1A primer** | **5’ Sequence** | **3’ Sequence** | **Size**  **(bp)** |
| --- | --- | --- | --- |
| 1 | AGACATCGCGGGGACCGA | CTGCCCGCCCTGGGCTC | 127 |
| 2 | GGCCTCTGTGATGAGGCTT | GAAGGTGGTCGCAATGTTTTG | 150 |
| 3b | CACAGATGATGGTGACATGAT | GATTTTATGATCTAGCCCTAATTT | 130 |
| 4 | CTCATCCATGTGACCATGAG | GTACTTACCCAAGGCAAAATG | 156 |
| 5 | CTCAGAATGAAGTGTACCCTA | GAATTGTGAGGGAAGGTTTAC | 160 |
| 6 | GCAAGACTTTCCTCAGTCGA | GAATATTCACCATAGCAAAGATT | 136 |
| 7 | ATGAATATTATCATGCTTTGGAC | GTACAGTTTGGCTACCCATCTA | 132 |
| 8 | GGATATGTCTGGGTTGAAACT | GGATACTCTGTTCATTTATAGG | 167 |
| 9 | TGCTGGCCCCAGCCGCT | TGCTACAAGCCCCATTTCAAC | 115 |
| 10 | GAACCAAATCCAGAGTCACTG | CAGAAAGGGACAACTTTCAGA | 142 |
| 11 | GTTGGAATTGGTAGAAAAACTTT | GTGACTTTGAGTTTCACTTGTT | 137 |
| 12 | CAGACAGAAAAATCTCATCCA | GTGATAGTACATGATTTTTAAACT | 140 |
| 13 | CAGAATGCTCAGAGAAAGCG | GTTTCCACGTTTCTTCCAAATA | 164 |
| 14 | TGCAAATCTAGTGAACAGAATG | CATGAAACATTTTTATTTAGGAGC | 124 |
| 15 | CACTAACTGTATTGTTTTGTTAC | CTTCAACACCTCCAACTCATA | 114 |
| 14/15 | GCTGAAGACACAGAAGCAAAG | CAATGGATGATGACTTCCAGT | 226/  100* |
| GAPDH | TGTTGCCATCAATGACCCCT | CTCCACGACGTACTCAGCG | 202 |

* : Before splicing process, the PCR products size is 226bp, after splicing, it is 100bp.
