# Supplementary material for: Mechanism of the natural product moracin-O derived MO-460 and its targeting protein hnRNPA2B1 on HIF-1α inhibition
Source: Exp Mol Med. 2019 Feb 12;51(2):10. doi: 10.1038/s12276-018-0200-4 (PMC6372683; doi:10.1038/s12276-018-0200-4)
Supplement: Supplementary file 3 — Supplementary Table S1 [file 12276_2018_200_MOESM3_ESM.docx]

**Supplementary Table S1.** Mascot search results: Original list of proteins detected as Biotin-MO-460 binding targets. The blue letter indicated proteins were MO-460 binding targets which have more than 30 % sequence coverage.

**Candidates (Cytosol)**

| **NCBI-GI** | **Identified proteins** | **Nominal  Mass (Mr)** | **Score** | **Queries  matched** | **Sequence  Coverage (%)** |
| --- | --- | --- | --- | --- | --- |
| *Nucleophosmin-related protein* | |  |  |  |  |
| 825671 | B23 nucleophosmin (280 AA) [Homo sapiens]  Nucleophospmin 3 | 31090 | 492 | 15 | 35 |
| 169172618 | similar to hCG2041218 [Homo sapiens] | 59042 | 119 | 8 | 12 |
|  |  |  |  |  |  |
| *Glyceraldehyde-3-phosphate dehydrogenase-related proteins* | |  |  |  |  |
| 7669492 | glyceraldehyde-3-phosphate dehydrogenase  [Homo sapiens] | 36201 | 305 | 16 | 39 |
| 230867 | Chain R, Twinning In Crystals Of Human Skeletal Muscle  D- Glyceraldehyde-3-Phosphate Dehydrogenase | 36024 | 172 | 8 | 12 |
|  |  |  |  |  |  |
| *Eukaryotic translation initiation factor-related poteins* | |  |  |  |  |
| 4503513 | eukaryotic translation initiation factor 3, subunit 2 beta,  36kDa [Homo sapiens] | 36878 | 162 | 5 | 17 |
| 38522 | human elongation factor-1-delta [Homo sapiens] | 31316 | 152 | 2 | 8 |
| 4758256 | eukaryotic translation initiation factor 2, subunit 1 alpha,  35kDa [Homo sapiens] | 36374 | 103 | 1 | 4 |
|  |  |  |  |  |  |
| *Others* | |  |  |  |  |
| 55956919 | heterogeneous nuclear ribonucleoprotein A/B isoform  a [Homo sapiens] | 36059 | 141 | 4 | 14 |
| 498910 | endothelial-monocyte activating polypeptide II | 34590 | 105 | 3 | 15 |
| 63101692 | Unknown (protein for IMAGE:4336253) [Homo sapiens] | 23902 | 65 | 4 | 13 |
| 114147469 | immunoglobulin heavy chain variable region  [Homo sapiens] | 12608 | 61 | 1 | 11 |
| 16552976 | unnamed protein product [Homo sapiens] | 81746 | 51 | 1 | 0 |
| 14141166 | poly(rC) binding protein 2 isoform b [Homo sapiens] | 38597 | 49 | 2 | 6 |
| 28336 | mutant beta-actin (beta'-actin) [Homo sapiens] | 42128 | 48 | 1 | 2 |
| 181914 | DNA-binding protein | 36086 | 46 | 1 | 2 |
| 1215669 | JTV-1 | 34787 | 44 | 1 | 2 |
| 13377630 | spermatid perinuclear RNA-binding protein  [Homo sapiens] | 74402 | 40 | 1 | 1 |
| 14719299 | HEJ1 [Homo sapiens] | 7522 | 38 | 1 | 15 |
| 340219 | vimentin | 53738 | 38 | 2 | 3 |
| 40788320 | KIAA0663 protein [Homo sapiens] | 90558 | 37 | 1 | 0 |
| 553734 | putative [Homo sapiens] | 2269 | 36 | 2 | 38 |
| 18676472 | FLJ00133 protein [Homo sapiens] | 144232 | 35 | 1 | 0 |
| 38051823 | Plasminogen [Homo sapiens] | 93263 | 35 | 1 | 1 |
| 58222127 | anti-tetanus toxoid immunoglobulin heavy chain variable region [Homo sapiens] | 13482 | 33 | 1 | 5 |
|  |  |  |  |  |  |
| *Aldolase-related proteins* | |  |  |  |  |
| 28614 | Aldolase A [Homo sapiens] | 39706 | 326 | 18 | 39 |
| 312137 | fructose bisphosphate aldolase [Homo sapiens] | 39816 | 117 | 5 | 11 |
|  |  |  |  |  |  |
| *Others* | |  |  |  |  |
| 2737886 | NAD+-specific isocitrate dehydrogenase beta  precursor [Homo sapiens] | 42470 | 162 | 3 | 9 |
| 460771 | hnRNP-E1 [Homo sapiens] | 38015 | 132 | 4 | 11 |
| 4758118 | death-associated protein 3 [Homo sapiens] | 45880 | 124 | 2 | 6 |
| 3986482 | translation initiation factor eIF3 p40 subunit; eIF3p40 [Homo sapiens] | 40075 | 80 | 5 | 12 |
| 12751096 | PNAS-125 [Homo sapiens] | 23853 | 63 | 1 | 5 |
| 10197644 | MDS023 [Homo sapiens] | 26714 | 61 | 2 | 7 |
| 6970062 | gastric-associated differentially-expressed protein  YA61P [Homo sapiens] | 48318 | 53 | 3 | 4 |
| 14719299 | HEJ1 [Homo sapiens] | 7522 | 48 | 1 | 15 |
| 3387977 | ABC transporter [Homo sapiens] | 57777 | 44 | 1 | 1 |
| 29126836 | MRPS9 protein [Homo sapiens] | 35297 | 43 | 2 | 7 |
| 181608 | desmoplakin | 202206 | 42 | 2 | 0 |
| 181608 | tau-tubulin kinase [Homo sapiens] | 183893 | 38 | 2 | 0 |
| 38051823 | Plasminogen [Homo sapiens] | 93263 | 37 | 1 | 1 |
| 114147469 | immunoglobulin heavy chain variable region  [Homo sapiens] | 12608 | 37 | 1 | 11 |
|  |  |  |  |  |  |
| *Nucleolin-related protein* | |  |  |  |  |
| 189306 | Nucleolin | 76355 | 997 | 30 | 34 |
|  |  |  |  |  |  |

| **Candidates (Nuclei)** | |  |  |  |  |
| --- | --- | --- | --- | --- | --- |
|  |  |  |  |  |  |
| **NCBI-GI** | **Identified proteins** | **Nominal Mass (Mr)** | **Score** | **Queries matched** | **Sequence Coverage (%)** |
| *hnRNP-related proteins* | |  |  |  |  |
| 133254 | heterogeneous nuclear ribonucleoprotein A1  [Homo sapiens] | 38822 | 591 | 20 | 32 |
| 4504447 | heterogeneous nuclear ribonucleoprotein A2/B1  isoform A2 [Homo sapiens] | 35984 | 212 | 10 | 24 |
| 1911429 | heterogeneous nuclear ribonucleoprotein  [human, placenta, Peptide, 305 aa] | 30882 | 95 | 4 | 9 |
| 119623231 | hCG1989619, isoform CRA_c [Homo sapiens] | 12552 | 41 | 3 | 12 |
| 34740329 | heterogeneous nuclear ribonucleoprotein A3  [Homo sapiens] | 39571 | 41 | 4 | 5 |
|  |  |  |  |  |  |
| *Others* | |  |  |  |  |
| 4885377 | histone cluster 1, H1d [Homo sapiens] | 22336 | 82 | 4 | 18 |
| 31092 | unnamed protein product [Homo sapiens] | 50095 | 73 | 3 | 5 |
| 72534660 | splicing factor, arginine/serine-rich 7 [Homo sapiens] | 27350 | 70 | 4 | 8 |
| 1431699 | Chain A, U1a MutantRNA COMPLEX + GLYCEROL | 11183 | 65 | 2 | 18 |
| 4502899 | clathrin, light polypeptide A isoform a [Homo sapiens] | 23647 | 65 | 2 | 7 |
| 16751921 | dermcidin preproprotein [Homo sapiens] | 11277 | 60 | 1 | 10 |
| 553724 | putative [Homo sapiens] | 2122 | 54 | 3 | 38 |
| 2460037 | m6A methyltransferase [Homo sapiens] | 64254 | 50 | 1 | 1 |
| 38479 | putative homeotic protein [Homo sapiens] | 41126 | 47 | 1 | 1 |
| 5031857 | L-lactate dehydrogenase A isoform 1 [Homo sapiens] | 36665 | 46 | 2 | 4 |
| 34529624 | unnamed protein product [Homo sapiens] | 72605 | 43 | 1 | 1 |
| 48717244 | zinc finger protein 782 [Homo sapiens] | 80852 | 43 | 1 | 0 |
| 14719299 | HEJ1 [Homo sapiens] | 7294 | 42 | 1 | 15 |
| 114147469 | immunoglobulin heavy chain variable region  [Homo sapiens] | 12494 | 39 | 1 | 11 |
| 340219 | vimentin | 53681 | 38 | 2 | 4 |
| 38051823 | Plasminogen [Homo sapiens] | 90526 | 34 | 1 | 1 |
|  |  |  |  |  |  |
| *hnRNP-related proteins* | |  |  |  |  |
| 14043072 | heterogeneous nuclear ribonucleoprotein A2/B1  isoform B1 [Homo sapiens] | 37407 | 380 | 14 | 33 |
| 14110407 | heterogeneous nuclear ribonucleoprotein D-like  [Homo sapiens] | 46409 | 93 | 2 | 5 |
| 14141157 | heterogeneous nuclear ribonucleoprotein H3 isoform  a [Homo sapiens] | 36903 | 91 | 3 | 7 |
| 34740329 | heterogeneous nuclear ribonucleoprotein A3  [Homo sapiens] | 39571 | 88 | 5 | 9 |
| 133254 | heterogeneous nuclear ribonucleoprotein A1 | 38822 | 40 | 5 | 9 |
| 1911429 | heterogeneous nuclear ribonucleoprotein  [human, placenta, Peptide, 305 aa] | 30882 | 37 | 2 | 2 |
|  |  |  |  |  |  |
| *Others* | |  |  |  |  |
| 28592 | serum albumin [Homo sapiens] | 69321 | 146 | 4 | 4 |
| 38522 | human elongation factor-1-delta [Homo sapiens] | 31202 | 79 | 2 | 7 |
| 4506005 | protein phosphatase 1, catalytic subunit, beta isoform 1 [Homo sapiens] | 37163 | 67 | 1 | 3 |
| 3334899 | autoantigen p542 [Homo sapiens] | 30420 | 63 | 3 | 8 |
| 16751921 | dermcidin preproprotein [Homo sapiens] | 11277 | 57 | 2 | 20 |
| 31092 | unnamed protein product [Homo sapiens] | 50095 | 53 | 1 | 2 |
| 1584035 | Lasp-1 protein | 29786 | 52 | 3 | 11 |
| 2460037 | m6A methyltransferase [Homo sapiens] | 64254 | 50 | 1 | 1 |
| 190238 | nucleolar phosphoprotein B23 | 9189 | 49 | 2 | 18 |
| 6677723 | replication factor C 5 isoform 1 [Homo sapiens] | 38472 | 49 | 1 | 3 |
| 13111899 | THOC6 protein [Homo sapiens] | 34827 | 45 | 1 | 3 |
| 114147469 | immunoglobulin heavy chain variable region [Homo sapiens] | 12494 | 45 | 1 | 11 |
| 553734 | putative [Homo sapiens] | 2212 | 44 | 1 | 38 |
| 14719299 | HEJ1 [Homo sapiens] | 7294 | 44 | 1 | 15 |
| 182592 | fibrillarin | 33797 | 36 | 1 | 3 |
| 119581910 | hCG1641252 [Homo sapiens] | 26332 | 36 | 1 | 4 |
|  |  |  |  |  |  |
| *non-POU domain containing, octamer-bindingprotein* | |  |  |  |  |
| 348239 | [Human mRNA, complete cds.], gene product | 54233 | 859 | 34 | 38 |
|  |  |  |  |  |  |
| *Others* | |  |  |  |  |
| 4506243 | polypyrimidine tract-binding protein 1 isoform a  [Homo sapiens] | 59596 | 84 | 6 | 7 |
| 31873900 | hypothetical protein [Homo sapiens] | 555302 | 82 | 3 | 7 |
| 2737894 | Cbf5p homolog | 57672 | 78 | 2 | 3 |
| 4508017 | zinc finger protein 207 isoform a [Homo sapiens] | 50717 | 78 | 3 | 6 |
| 16751921 | dermcidin preproprotein [Homo sapiens] | 11277 | 75 | 2 | 20 |
| 1200184 | stimulator of TAR RNA binding | 57803 | 68 | 1 | 2 |
| 226021 | growth regulated nuclear 68 protein | 66881 | 64 | 1 | 2 |
| 7023323 | unnamed protein product [Homo sapiens] | 41713 | 56 | 4 | 10 |
| 460789 | transformation upregulated nuclear protein  [Homo sapiens] | 51040 | 51 | 2 | 5 |
| 114147469 | immunoglobulin heavy chain variable region  [Homo sapiens] | 12494 | 49 | 1 | 11 |
| 1657698 | hyaluronan receptor [Homo sapiens] | 84146 | 43 | 1 | 0 |
| 2708305 | U4/U6 small nuclear ribonucleoprotein hPrp4 [Homo sapiens] | 58431 | 41 | 1 | 1 |
| 35505 | pyruvate kinase [Homo sapiens] | 57841 | 41 | 1 | 2 |
| 14719299 | HEJ1 [Homo sapiens] | 7294 | 41 | 1 | 15 |
| 1770368 | put. B7,3 molecule of CD80-CD86 family  [Homo sapiens] | 33797 | 41 | 1 | 1 |
| 6650606 | glutaminase [Homo sapiens] | 66267 | 41 | 1 | 1 |
| 157881246 | Chain A, Solution Structure Of Polypyrimidine Tract  Binding Protein Rbd1 Complexed With Cucucu Rna | 13072 | 37 | 2 | 16 |
| 10863997 | polypyrimidine tract binding protein 2  [Homo sapiens] | 57455 | 37 | 1 | 1 |
| 553734 | putative [Homo sapiens] | 2212 | 37 | 2 | 38 |
| 11128033 | protocadherin gamma subfamily B,  3 isoform 1 precursor [Homo sapiens] | 101170 | 37 | 1 | 0 |
| 7020479 | unnamed protein product [Homo sapiens] | 45865 | 36 | 2 | 2 |
| 551450 | splicing factor SF3a60 [Homo sapiens] | 58740 | 36 | 2 | 3 |
|  |  |  |  |  |  |
| *Heat shock-related proteins* | |  |  |  |  |
| 6807647 | hypothetical protein [Homo sapiens],  Heat shock protein HSP 90 beta | 85189 | 905 | 34 | 38 |
| 83699649 | heat shock 90kDa protein 1, alpha [Homo sapiens] | 98652 | 542 | 26 | 26 |
| 61104911 | heat shock protein 90Bb [Homo sapiens] | 49377 | 259 | 11 | 17 |
| 74722491 | Putative heat shock protein HSP 90-beta 4 | 58855 | 93 | 5 | 8 |
|  |  |  |  |  |  |
| *Keratin-related proteins* | |  |  |  |  |
| 11935049 | keratin 1 [Homo sapiens] | 66198 | 446 | 14 | 19 |
| 547754 | Keratin, type II cytoskeletal 2 epidermal | 66110 | 222 | 5 | 8 |
| 55956899 | keratin 9 [Homo sapiens] | 62255 | 214 | 6 | 10 |
| 28317 | unnamed protein product [Homo sapiens] | 59720 | 121 | 6 | 6 |
| 119617032 | keratin 6B, isoform CRA_a [Homo sapiens] | 60159 | 115 | 4 | 6 |
| 186685 | keratin type 16 | 51010 | 52 | 7 | 8 |
|  |  |  |  |  |  |
| *Others* | |  |  |  |  |
| 6005942 | valosin-containing protein [Homo sapiens] | 89950 | 512 | 21 | 23 |
| 189306 | nucleolin | 76355 | 287 | 10 | 11 |
| 4507241 | structure specific recognition protein 1  [Homo sapiens] | 81367 | 264 | 8 | 13 |
| 179832 | calnexin | 67948 | 229 | 9 | 13 |
| 4503483 | eukaryotic translation elongation factor 2  [Homo sapiens] | 96246 | 222 | 9 | 10 |
| 340219 | vimentin | 53738 | 116 | 3 | 4 |
| 182855 | 80K-H protein | 60228 | 110 | 4 | 7 |
| 1082886 | tumor necrosis factor type 1 receptor associated  protein TRAP-1 - human | 75694 | 98 | 1 | 2 |
| 28336 | mutant beta-actin (beta'-actin) [Homo sapiens] | 42128 | 92 | 3 | 8 |
| 4826960 | glutaminyl-tRNA synthetase [Homo sapiens] | 88655 | 79 | 2 | 2 |
| 7023756 | unnamed protein product [Homo sapiens] | 54731 | 69 | 1 | 2 |
| 6424942 | ALG-2 interacting protein 1 [Homo sapiens] | 96646 | 68 | 1 | 1 |
| 5107666 | Chain A, Structure Of Importin Beta  Bound To The Ibb Domain Of Importin Alpha | 98484 | 67 | 1 | 1 |
| 169213772 | similar to actin alpha 1 skeletal muscle protein  [Homo sapiens] | 51513 | 64 | 2 | 2 |
| 177207 | 4F2 antigen heavy chain | 58049 | 61 | 1 | 2 |
| 1232079 | huMCM5 [Homo sapiens] | 82578 | 54 | 2 | 3 |
| 252585 | interferon-stimulated gene factor 3 alpha 91/84 kda  protein | 11821 | 51 | 1 | 11 |
| 11559925 | X-prolyl aminopeptidase (aminopeptidase P) 3,  putative [Homo sapiens] | 57624 | 49 | 1 | 1 |
| 553734 | putative [Homo sapiens] | 2269 | 48 | 1 | 38 |
| 755746 | p85Mcm protein [Homo sapiens] | 69791 | 46 | 1 | 3 |
| 49065664 | katanin p60 subunit A-like 2 [Homo sapiens] | 53003 | 43 | 1 | 2 |
| 37433 | unnamed protein product [Homo sapiens] | 85304 | 41 | 1 | 1 |
| 62420949 | actin-like protein [Homo sapiens] | 11568 | 40 | 1 | 17 |
| 119629468 | hCG2011852 [Homo sapiens] | 790386 | 39 | 2 | 0 |
| 38051823 | Plasminogen [Homo sapiens] | 93263 | 38 | 2 | 1 |
|  |  |  |  |  |  |
| *helicase related proteins* | |  |  |  |  |
| 17402900 | Far upstream element-binding protein 1 | 67518 | 504 | 28 | 30 |
| 47678395 | DDX17 | 72511 | 362 | 15 | 17 |
|  |  |  |  |  |  |
| *hnRNP-related proteins* | |  |  |  |  |
| 228008291 | heterogenous nuclear ribonucleoprotein Q isoform | 69590 | 251 | 12 | 15 |
| 5454064 | RNA-binding protein 14 | 69449 | 215 | 14 | 13 |
| 5031753 | Heterogenous nuclear ribonucleoprotein H1 | 49198 | 222 | 15 | 16 |
| 48145673 | hnRNPH1 | 49099 | 222 | 13 | 16 |
| 119631468 | heterogenous nuclear ribonucleoprotein A3 isoform | 37006 | 218 | 12 | 14 |
| 55956916 | heterogenous nuclear ribonucleoprotein A/B isoform | 35945 | 155 | 8 | 10 |
|  |  |  |  |  |  |
| *Others* | |  |  |  |  |
| 194388148 | unnamed protein products | 34168 | 218 | 12 | 15 |
| 189069313 | unnamed protein products | 69559 | 251 | 11 | 15 |
