# Supplementary figures and images for: Mechanism of the natural product moracin-O derived MO-460 and its targeting protein hnRNPA2B1 on HIF-1α inhibition
Source: Exp Mol Med. 2019 Feb 12;51(2):10. doi: 10.1038/s12276-018-0200-4 (PMC6372683; doi:10.1038/s12276-018-0200-4)

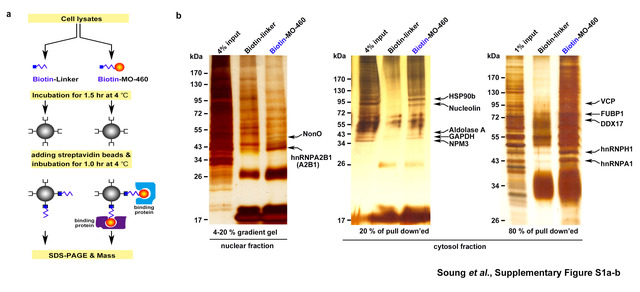

Supplement: Supplementary file 4 — Supplementary Figure S1 [file 12276_2018_200_MOESM4_ESM.jpg]

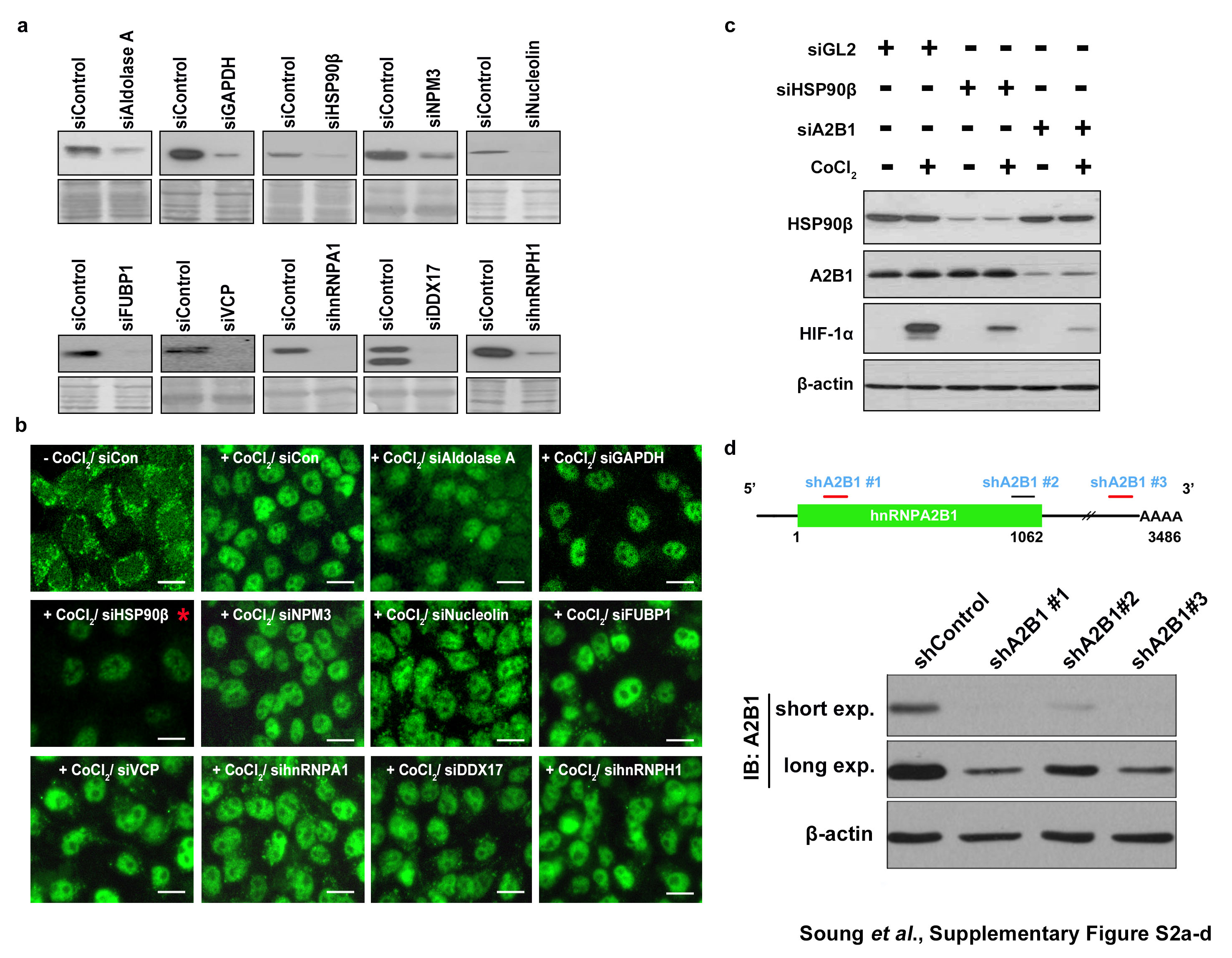

Supplement: Supplementary file 5 — Supplementary Figure S2 [file 12276_2018_200_MOESM5_ESM.jpg]

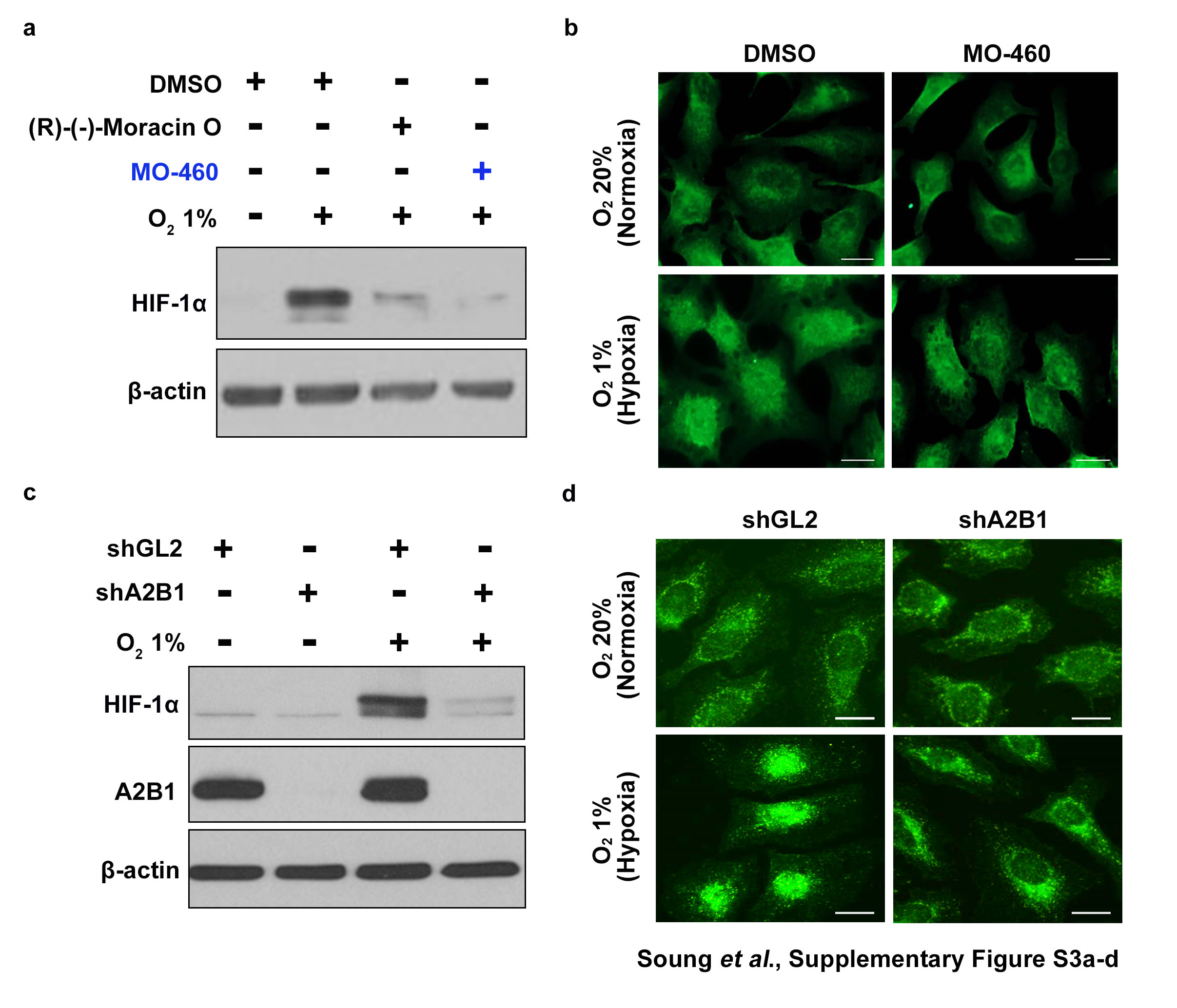

Supplement: Supplementary file 6 — Supplementary Figure S3 [file 12276_2018_200_MOESM6_ESM.jpg]

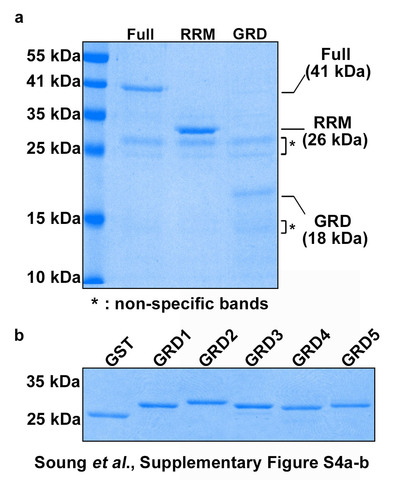

Supplement: Supplementary file 7 — Supplementary Figure S4 [file 12276_2018_200_MOESM7_ESM.jpg]

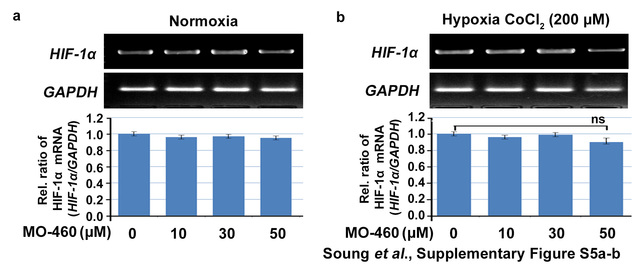

Supplement: Supplementary file 8 — Supplementary Figure S5 [file 12276_2018_200_MOESM8_ESM.jpg]

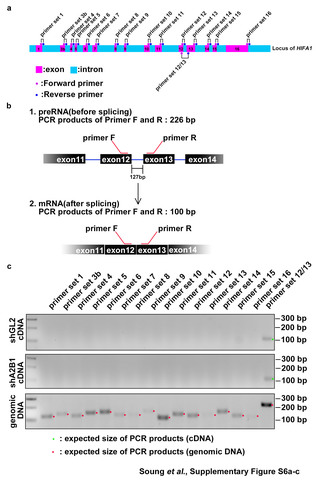

Supplement: Supplementary file 9 — Supplementary Figure S6 [file 12276_2018_200_MOESM9_ESM.jpg]

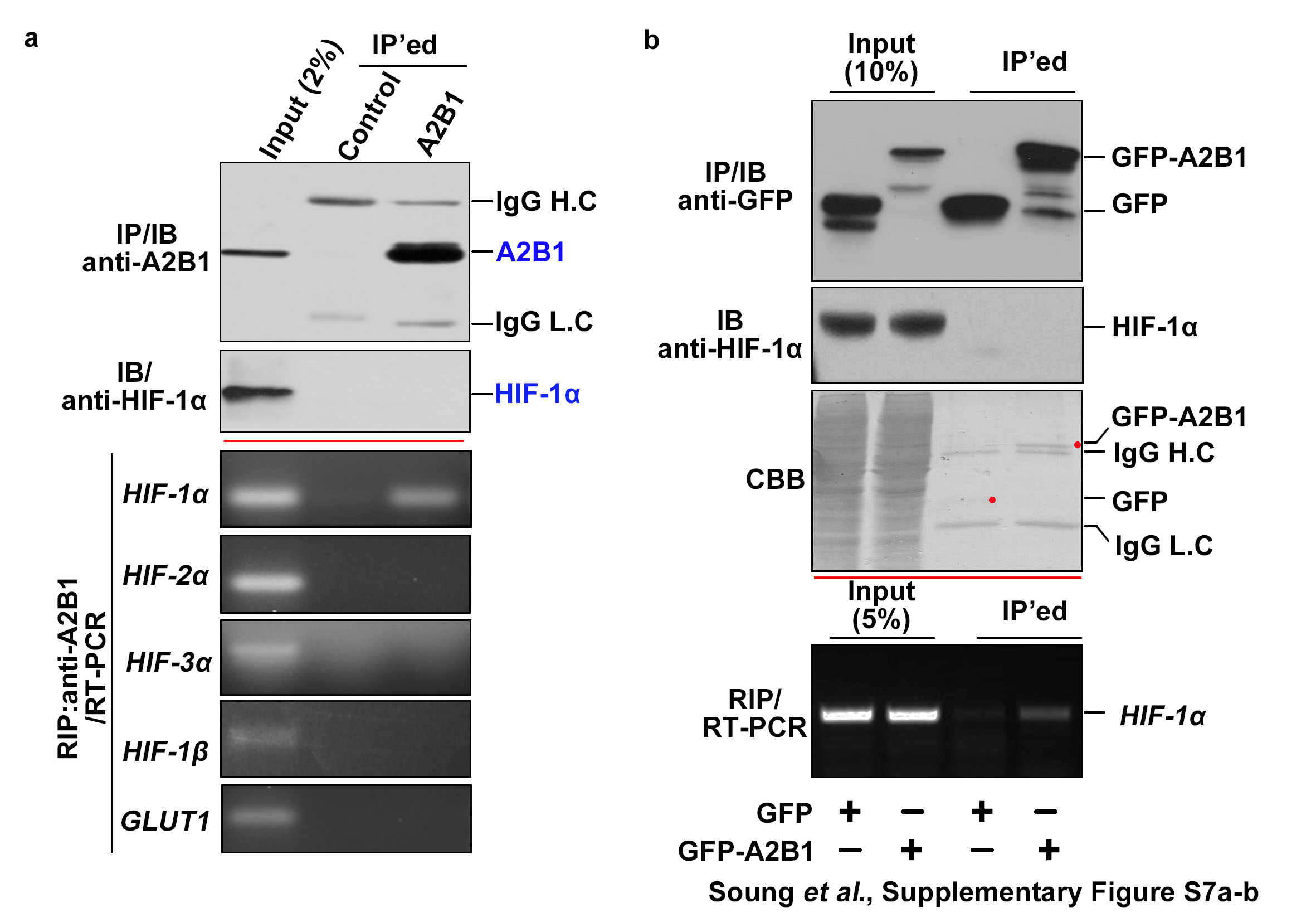

Supplement: Supplementary file 10 — Supplementary Figure S7 [file 12276_2018_200_MOESM10_ESM.jpg]

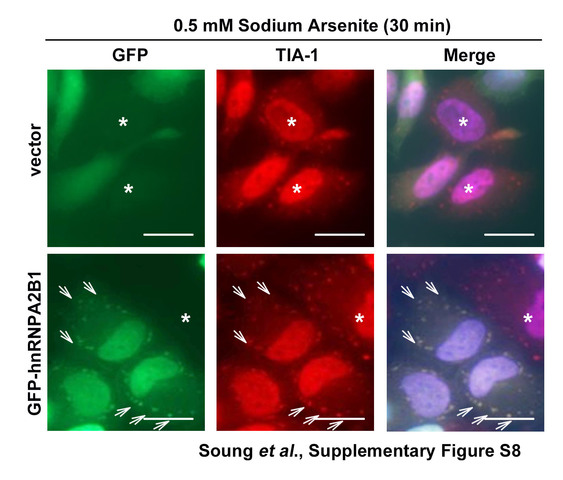

Supplement: Supplementary file 11 — Supplementary Figure S8 [file 12276_2018_200_MOESM11_ESM.jpg]
